# Effectiveness of an unguided modular online intervention for highly anxious parents in preventing anxiety in their children: a parallel group randomised controlled trial

**DOI:** 10.1016/j.lanepe.2024.101038

**Published:** 2024-09-04

**Authors:** Abby Dunn, James Alvarez, Amy Arbon, Stephen Bremner, Chloe Elsby-Pearson, Richard Emsley, Christopher Jones, Peter Lawrence, Kathryn J. Lester, Natalie Morson, Julia Simner, Abigail Thomson, Sam Cartwright-Hatton

**Affiliations:** a University of Sussex, Falmer, United Kingdom; b University Hospitals Sussex NHS Foundation Trust, Brighton, United Kingdom; c Brighton and Sussex Medical School, Brighton, United Kingdom; d King's College London, London, United Kingdom; e University of Southampton, Southampton, United Kingdom

**Keywords:** Anxiety, Child anxiety, Digital health, Parenting, Randomised controlled trial

## Abstract

**Background:**

Children whose parents have anxiety problems are at increased risk of developing anxiety themselves. Parenting behaviors are a contributing factor to intergenerational transmission. Interventions which seek to limit anxiogenic parenting behaviors have shown potential in reducing anxiety in offspring but are not widely accessible. This prevention trial aimed to establish the effectiveness of an unguided modular online intervention for highly anxious parents in preventing anxiety in their children.

**Methods:**

A parallel group, block-randomised controlled trial of unblinded participants in a 1:1 ratio was conducted to compare efficacy of the online course compared to a no-intervention control. The intervention comprised 8 modules, of approximately 20 min each, and participants progressed through the course at their own pace. The study was conducted entirely online with a self-referred UK-based community sample of parents (child 2–11 years) with substantial anxiety. The primary outcome measure was change in parent-reported child anxiety, as measured by the Spence Children's Anxiety Scale–Parent Report (SCAS-P) or Spence Pre-School Anxiety Scale–Parent Report (Preschool SCAS). Secondary outcomes were child internalising, externalising, and attentional symptoms (Pediatric Symptom Checklist), and parent anxiety (SCARED-Adult). Analyses using complete case analysis following intention to treat principles investigated intervention effects at 6 months (primary analysis) and additionally at 9 to 25-months’ follow-up. Trial registration: ClinicalTrials.GovNCT04755933, https://clinicaltrials.gov/ct2/show/NCT04755933.

**Findings:**

1811 participants (intervention = 900; control = 911; 92.7% (1677/1810) female; 85.3% (1535/1800) White-British; 66.8% (1201/1799) university educated). Participant retention (based on primary outcome completion) at T2 (6-months post consent) was 67.6% overall (n = 1224) and substantially lower in the intervention arm 57.3% ((516/900) control = 77.8% (708/910)). Child anxiety was lower in the intervention group compared to control at 6-month follow-up (adjusted effect size estimate −0.15 (95% CI: −0.23 to −0.08, p < 0.001). There was very strong evidence that those in the intervention arm had lowered child anxiety (standardised SCAS score) compared to the control arm, with an effect size (Cohen's d) of −0.16 (95% CI: −0.23 to −0.08, p < 0.001). The difference in standardised Spence Child Anxiety Scale score between the arms was −0.15 standard deviations. On the original scales for SCAS-P (0–114) and Preschool SCAS (0–112), this corresponds to a reduction of −2.38 (95% CI: −3.59 to −1.16) and −2.68 (95% CI: −4.05 to −1.31), respectively. No reported harms.

**Interpretation:**

A clinically unsupported online intervention designed for parents with high levels of anxiety is effective in reducing anxiety and internalising symptoms in their children, and also anxiety in parents. Given the low resource intensity of this intervention, and the positive effects reported here, these findings suggest it has promise in limiting the intergenerational transmission of severe anxiety.

**Funding:**

This work was supported by 10.13039/100019647Kavli Trust (grant 38/19).

**Research in context**

**Evidence before this study**

Two systematic reviews informed the project. The first searched Cochrane Systematic Reviews and the Cochrane Register of Controlled Trials, PsycINFO, Medline, Education Resources Information Centre (search Feb 2021) with inclusion criteria: participants were parents with psychiatric illness or parents at high risk of psychiatric illness (e.g., homeless parents); intervention effect size reported; minimum one child outcome measure reported; published in a peer reviewed journal and written in English. Search terms included [Intervention OR Treatment OR Therapy] AND [Parents OR Parenting] AND [mental disorders]. Of 127 included papers, two studies were focused on parents with anxiety.^1^ The studies were of fair and good however both were therapist-led face-to-face interventions of which one was oriented to the treatment of children with a pre-existing diagnosis of anxiety rather than prevention. The second paper, Ginsburg's (2009) small-sample pilot RCT found some evidence of the preventative benefit of intervention for parents who have anxiety disorders. Two papers (which were not included in this review) were identified by the author of the current manuscript. A subsequent larger (N = 136) RCT by Ginsburg replicated earlier findings with 26% lower incidence of child anxiety in the intervention group with difference maintained at one-year follow-up. Cartwright-Hatton's face-to-face intervention identified a reduction in child anxiety (16.5% fewer diagnoses in the intervention arm at 12-month follow-up, compared to the control group). The second systematic review searched PsychInfo, EMBASE, PubMed, CINAHL, and Google Scholar in December 2012 with inclusion criteria: studies published after January 1990 focused on children and young people (<25 years) or parents of children with mental health problems; internet-based interventions targeting anxiety; parallel, pre-post and observation studies with outcome measure of anxiety and/or depression diagnosis or severity.^2^ Studies were excluded if they were not published in English. Search terms focused on electronic provision (e.g., eHealth); mental health services; and children, young people and young adults. Six moderate or strong quality studies were included in the analysis. These interventions were highly effective; pooled effect sizes for intervention versus inactive control were −0.52 [95% CI: −0.90 to 0.14]).

**Added value of this study**

This study is the first known randomized controlled trial of an online intervention designed to limit the intergenerational transmission of anxiety. It extends the earlier findings from the face-to-face interventions with the evaluation of a lower-resource-intensive, and more flexible intervention, conducted with an extremely large sample size. In doing so it has generated clear evidence that an online intervention delivered to parents with anxiety can reduce anxiety in their children.

**Implications of all the available evidence**

With efficacy well-established, the next step is to increase access to this preventive tool as a mechanism to reduce the levels of anxiety in children and young people. This requires investigation into mechanisms through which user engagement can be increased, and into effective approaches for embedding the intervention within mental health and other service contexts.

## Introduction

Anxiety disorders are the second most common mental illness in British adults, with 37% of women and 30% of men experiencing high levels of anxiety at any time.^3^ This equates to 8.2 million people in the UK with a probable anxiety disorder, globally numbering 374 million people.^4^ Childhood anxiety is also highly prevalent: one in six children aged under 16 years meet criteria for an emotional disorder (anxiety, obsessive compulsive disorder (OCD), phobias, depression) with anxiety the most common mental health condition of pre-adolescence.^5^^,^^6^ Furthermore, anxiety in children and young people is associated with impaired educational, employment and health outcomes, including elevated risk of developing mood disorders and substance abuse in adulthood. Childhood anxiety is costly, with societal costs of £4679 per child per annum.^7^ The extent of anxiety disorders amongst the young is partially explained by high rates of intergenerational transmission: put simply, anxiety runs in families. A child whose mother has an anxiety disorder is twice as likely to develop their own anxiety disorder than a peer whose mother does not.^8^ This intergenerational transmission is driven by the interplay of inherited biological factors and environmental influences, of which parents are a major contributor (see Murray, 2009^9^). A body of evidence implicates a range of anxiogenic parenting behaviours, such as modelling of fear and encouraging threat avoidance, in this transmission.^10^ This association between parenting behaviours and child symptoms generates a clear target for preventive intervention.

Psychological interventions that seek to limit the transmission of anxiety from parent to child have generated promising results^11^^,^^12^: Cartwright-Hatton and colleagues designed a brief, group-based intervention for parents with anxiety disorders. This intervention was focused on supporting consistent and positive behaviour management approaches and discouraging parents' and children's threat avoidance. In a randomised controlled trial, children whose parents had received the intervention were 16.5% less likely to have an anxiety disorder one year later, compared to control children.^12^ While promising, solely delivering this intervention within health services limits its reach to the approximately 25% of people with a probable anxiety disorder (in the UK) who receive treatment.^13^ [NO_PRINTED_FORM]It also excludes those who cannot attend because of childcare or work commitments, or because they are too anxious to attend a group. Given the high and rising prevalence of severe anxiety, the inequalities in access to mental health services, and the clear risk parental anxiety poses, parental anxiety should be viewed as a public health problem. Therefore, interventions that can be delivered flexibly, at the point of need and, ideally, without need for clinical input, are urgently required.^14^

The recent conditional recommendation of online interventions (that show early evidence of value) for anxiety and depression by the National Institute for Health and Care Excellence (NICE), the widespread National Health Service (NHS) recommendation of web/smartphone apps, and the foregrounding of integrated (face-to-face and digital) intervention by the NHS Transformation Directorate, all point to a growing role for digitally-based support for mental health.^15^, ^16^, ^17^, ^18^ There are clear signals that the acceptability and efficacy of online-delivered interventions can meet the standards of those delivered face-to-face.^19^ The Parenting with Anxiety project sought to engage with this landscape by developing a preventive online intervention focused on supporting parents who are highly anxious to limit anxiety in their children. This modular, online course was an adaptation of Cartwright-Hatton's face-to-face group-based intervention. This digital intervention was designed to be accessed by parents in their own time, without any clinical input. This was evaluated via a community-based randomised controlled trial, wherein, although all participants experienced high levels of anxiety, they were not required to be in contact with mental health services. The goal was to evaluate the utility and efficacy of this inexpensive, highly accessible intervention in managing a growing public health problem.

The prevention study had four objectives: 1) Investigate the effectiveness of a digital intervention aimed at anxious parents and designed to prevent anxiety in their children. 2) Test whether these effects were moderated by participant characteristics. 3) Determine which intervention modules had most/least impact on outcomes. 4) Explore the impact of co-parent anxiety and parenting behaviours on child outcomes. Objectives 3 and 4 will be reported elsewhere. We hypothesised that children of parents allocated to the intervention arm would have lower anxiety at follow-up than children whose parents were allocated to the control arm.

## Methods

### Study design

A parallel groups Randomised Controlled Trial with two equal-sized arms. The study was completed entirely online, with all participants self-referred from the community. The study was sponsored by the University of Sussex and approved by the Cross Schools Research Ethics Committee (ER/SC430/1). It is registered at ClinicalTrials.gov (NCT04755933, https://clinicaltrials.gov/ct2/show/NCT04755933). The protocol is available at https://doi.org/10.2196/40707.

### Participants

#### Eligibility

The study employed minimal exclusion criteria to reflect a putative eventual community implementation. No exclusions were made on grounds of psychological, neuropsychological (e.g., attention-deficit hyperactivity disorder (ADHD), autism) or physical conditions, or prior treatment, in either parent or child. All participants self-identified as follows.

• UK resident, aged 16+
• Subjective, self-reported, substantial levels of current/lifetime anxiety [*Would you describe yourself as high in anxiety? This means you feel your anxiety is getting in the way of living your life as you would like to. This may have been something you experienced before the COVID-19 crisis or you may have noticed high levels of persistent anxiety in the period since March 2020*]. It was not necessary to have a diagnosed/diagnosable anxiety disorder.
• Parent (any gender, adoptive/biological/step/foster/grandparent) of a child aged 2–11 years, with whom they had sufficient contact for at least 50 days per year to be able to report on the child's anxiety level.
• Willing to commit to completing follow-up measures, regardless of intervention arm allocation.

Participants were also invited to optionally nominate a ‘co-respondent’ (e.g., childcare provider, the other parent, grandparent, close family friend) to complete some basic outcome measures about the index child, and when nominated, this co-respondent was offered a small financial incentive to return data. For around half of participants, the parent was also offered an additional small financial incentive if their co-respondent returned data (the effect of this additional incentive is explored in a paper reported elsewhere https://doi.org/10.1016/j.conctc.2023.101090). It was not expected that this would yield sufficient co-respondent data to conduct an appropriately powered analysis of the effect of the intervention. However, it was included to allow the researchers to begin to understand the nature and size of any biases in reporting when highly anxious parents are asked to report on their child's anxiety, and the results of these investigations will be reported elsewhere.

#### Recruitment

Participants were self-referred and were recruited using a range of community-based approaches, including advertisements in print/social media, third sector organisations and the education sector. The two biggest sources of recruitment were via the Genetic Links to Anxiety and Depression (GLAD) study within the National Institute for Health and Care Research (NIHR) Mental Health Bioresource, and via advertisements in magazines issued free of charge to parents of primary school age children (4–11 years). Participants received study information, self-screened for eligibility, provided informed consent, were invited to refer a co-respondent (to provide some additional data on the child), and completed baseline measures, all remotely and online, prior to randomisation.

### Randomization and masking

Participants were randomised to the intervention or control arm using block randomisation, (randomly varied block sizes in multiples of 4 up to 20), from predefined lists generated by the Brighton and Sussex Clinical Trials Unit. Given the large sample size, stratified randomisation was deemed unnecessary. Participants were randomised once consent was granted but were informed of their allocation only after they had completed baseline (T1) questionnaires. To facilitate analysis of individual intervention module effects, participants in the intervention arm were further randomised into one of eight conditions, determining which eight (of nine possible) intervention modules were presented to them. Parents were aware of their allocation to the main intervention or control arm, but not of this further randomisation of modules in the intervention arm. Due to the self-report data collection and digital, unguided intervention delivery, there was minimal risk of bias in outcome assessment.

### Procedures

#### Study arms

##### Online course

The online intervention mirrored the content and format of the evidence-based, face-to-face Raising Confident Children Course.^12^ It is rooted primarily in cognitive behavioural and social learning approaches which are employed to help parents to develop a clear, calm approach to managing children's behaviour (including anxious behaviour) and to be aware of any of their own parenting behaviour that might be anxiogenic. See Table 1 for an overview of content. The course comprised one ‘core’ module, which all parents in the intervention arm were invited to complete, followed by a further eight modules, of which participants were given access to seven (one module disabled at random to facilitate a component analysis of module efficacy [study objective 3, to be reported elsewhere]). The order in which the seven additional modules were listed to each participant was randomised, but participants could access all from the outset, and could complete them in any order. Each module contained video, animated and written content, alongside activities such as quizzes and action planning. Modules focused on one topic and offered positively framed, non-stigmatising information and tools to facilitate parenting behaviour change. Each module took approximately 20–30 min to complete, and participants were encouraged to complete each module and accompanying home tasks before moving onto the next. No clinical support was provided to parents at any point, (although technical support was available by email). However, participants could optionally share a copy of the intervention (e.g., with a co-parent) to support their learning. Email and SMS ‘nudges’ (up to three) were issued to participants who had been randomised to the intervention but failed to sign up, or who disengaged (as defined by failure to login for more than seven days).

**Table 1:** Intervention module content.

Topic	Content
Core Module	All about anxiety and confidence in children
Module A	The role of avoidance and small steps to reducing it
Module B	Using play to develop children's confidence
Module C	Using ‘Emotion Coaching’ with children
Module D	Managing difficult behaviour: praise and reward
Module E	The role of sleep, exercise and diet in children's mental health
Module F	Parenting Hotspots: reducing overprotection
Module G	Modelling confident behaviour: compensating for parenting gaps
Module H	Managing difficult behaviour: consequences and limit setting

##### Control condition

Participants in the control condition received no intervention. As they were recruited from the community, without clinical referral, no instructions were given regarding accessing or continuing treatment for anxiety.

### Outcomes

Outcome measures were completed online by the parent at baseline (T1), and six months post consent (T2). To maximise longitudinal follow-up, a third online follow-up round was conducted at a fixed time-point (April 2023) which ranged from 9 to 25 months post consent. Participants who joined near the end of the study and had only recently completed T2 measures (less than 2 months previously) were excluded from T3. Median (interquartile range) time from consent for each arm to T3 was as follows: intervention arm: 14.2 (10.7–17.2) months; control arm: 14.2 (10.8–17.2) months; overall: 14.2 (10.8–17.2) months. For T3, an extended follow-up window was employed for two reasons: First, this allowed us to maximise the number of participants who were included in a longer-term follow up; a fixed window for the final follow up would have required a much longer and more costly study. Second, this extended window was planned to allow a more granular exploratory analysis of changes in outcomes over time which we plan to carry out for future publication. Participants received a £15 voucher on completion of each of T2 measures and T3 measures.

If a parent had more than one child within the target age-range, the platform allocated one ‘index’ child at random, who the parent then reported on for outcome measures at all time points.

#### Primary outcome

Change in parent-reported child anxiety: Spence Children's Anxiety Scale—Parent Report (SCAS-P^20^) assessment of anxiety in children aged five and above, or the Spence Pre-School Anxiety Scale—Parent Report (Preschool SCAS^21^) which is an adaptation of the SCAS-P for children aged under five. The SCAS is widely used and provides a measure of overall anxiety severity plus anxiety sub-types. It has good properties of validity, reliability, and acceptability to parents.^22^ The SCAS-P comprises 39 items which are scored on four-point scale (0 = never, 1 = sometimes, 2 = often, 3 = always). Preschool SCAS comprises 28 items rated on a five-point scale (0 = not true at all, 1 = seldom true, 2 = sometimes true, 3 = quite often true, 4 = very often true). Higher scores indicate greater severity of anxiety symptoms.

#### Secondary outcomes

##### Child outcomes

Child emotional and behavioural symptoms: The Pediatric Symptom Checklist (PSC-17^23^) is a 17-item general mental health screening tool. It comprises three subscales (internalizing, externalizing, attentional symptoms), rated by frequency (0 = never, 1 = sometimes, 2 = often). Higher scores indicate greater levels of symptoms. It has good validity and sensitivity with comparable case detection to semi-structured interviews and is widely used as a screening tool in primary care.

Child health: EuroQol Group's 5-dimensional proxy report–Youth version (EQ-5D-Y).^24^ This is a five-dimension, three non-numerical level, parent-report measure of child health-related quality of life, and a 0–100 scale (worst-best) measure of health on the given day. This was included to assist in economic analysis of the intervention and is not reported here.

##### Parent anxiety

SCARED-Adult (SCARED-A^25^): an adaptation of the widely-used SCARED measure of child anxiety. With 71 items, each rated for frequency (0 = almost never; 1 = sometimes; 2 = often) it assesses symptoms of anxiety disorders (panic disorder, generalised anxiety disorder, social phobia, separation anxiety disorder, obsessive compulsive disorder, post-traumatic stress disorder, phobias). Higher scores indicate greater severity of anxiety symptoms. It has good internal consistency and is highly correlated with results from the ADIS-IV-L diagnostic interview schedule.^25^

##### Parents’ wellbeing

Short Warwick Edinburgh Mental Wellbeing Scale (SWEMWBS^26^): a seven-item self-report measure (1 = none of the time, 2 = rarely, 3 = some of the time, 4 = often, 5 = all of the time), of positive mental health (positive affect, interpersonal relationships, positive functioning). Higher scores indicate greater levels of positive mental wellbeing. It is highly correlated with the original 14-item scale (WEMWBS) and has high internal consistency and good validity. This outcome is being used in analysis for a subsequent paper and is not reported here.

##### Participation distress questionnaire

Eight-item instrument designed by the study team to identity negative emotional impact caused by participating in the study.

### Other measures

#### Mediator variables (parenting behaviours)

Comprehensive Parenting Behaviour Questionnaire (CPBQ)^10^: A self-report instrument measuring parenting behaviours associated with risk of child anxiety. The original scale authors generated a psychometrically valid shortened version for use in the present study, comprising the following subscales: challenging behaviour; overinvolvement; warmth; negativity; negative discipline; positive discipline. For the few areas in our intervention not covered by the CPBQ (e.g., sleep, exercise, diet), items were identified from existing validated instruments or, in some cases, constructed for this study (full list of mediators can be accessed in published protocol^27^).

#### Demographic information

Parent and child gender, child age, previous parent or child anxiety treatment, and child developmental disabilities were assessed at baseline.

#### Intervention engagement

Data on participant engagement with the intervention was collected for all participants in the intervention arm. This recorded participant activities including signing-in to the intervention and number of modules/module components started and completed.

### Statistical analysis

#### Sample size

Based on our existing research, and because reduced effect sizes are expected in prevention trials, a small effect size was anticipated (Cohen's d = 0.2) which, with 90% power for 5% significance, required 526 participants in each trial arm.^28^ Allowing for 40% attrition, (typical for online trials with self-enrolment and longer-term follow-up),^29^ 877 participants were needed in each arm. Therefore, a total sample of 1754 participants was required.

All analyses were conducted according to the statistical analytical plan (SAP [v2 signed 7.7.23], see Supplementary Materials) using complete case analysis following intention to treat (ITT) principles (all randomised participants are included in analysis regardless of engagement with the intervention). The SAP was drafted by an independent, unblinded statistician, who had access to the outcome data. Missing data were prorated in accordance with the scale guidance where available. Where guidance was not available, data were prorated if ≥80% of the subscale/scale was complete, replacing missing items with the mean of that item for all available data from other participants, rounded to the nearest integer. Summary statistics (appropriate to the distribution) were used to describe the sample at baseline by arm and overall. Standardized z-scores were calculated for SCAS-P and Preschool SCAS (as appropriate), to allow combining of these scales (henceforth simply referred to as ‘SCAS’), which consist of different numbers of items and have different response keys.

#### Outcomes at time 2 and time 3

To investigate the effectiveness of the intervention on our primary outcome (child anxiety: SCAS), a multiple linear regression model was fitted for standardised SCAS score at T2, adjusting for covariates that were expected a priori to be prognostic of outcome: SCAS score at T1 (baseline), parent anxiety (SCARED-A overall score) at T1, parent birth gender, child gender and child age. Potential moderators of intervention effect (e.g., child anxiety severity) were assessed by repeating each model including intervention arm by moderator interactions as fixed effects. The full list of moderators assessed are reported in the SAP. Models were extended to include a random effect for participant to include T3 data.

For secondary outcomes, multiple linear regression models were used to assess the effect of the intervention on the following at T2 and T3: PSC internalising, PSC attention, PSC externalising and SCARED-A overall (parent anxiety). Each model included a fixed effect for intervention arm and for the following covariates, chosen a priori, that we considered could be prognostic of outcome: outcome at baseline, child anxiety at baseline (SCAS), parent anxiety (SCARED-A), parent gender, child gender at birth, child age. Each model was extended to include scores at T2 and T3.

#### Additional analyses

##### Complier Average Causal Effect (CACE)

Engagement with online interventions is typically highly variable. To examine the impact of engagement on outcomes, a CACE analysis was conducted. This modelled the effect of the intervention on participants in the intervention arm who complied with the intervention and those in control arm who, it was estimated, would have complied with the intervention if they had had the opportunity to do so. Three models were fitted, examining outcomes for three increasingly stringent definitions of complier: 1) those who began two modules, 2) those who completed two modules or more, 3) those who completed all eight modules. Module ‘completion’ was defined as having accessed at least 90% of a module's content.

##### Sensitivity analyses

Sensitivity analyses were performed to assess the robustness of conclusions to assumptions about the missing data, as primary analyses were conducted using complete cases. These were carried out by refitting the primary outcome model using full information maximum likelihood (FIML, to include all participants) and using Stata module RCTMISS to assess the effect of departures from the Missing at Random assumption in the context of differential attrition.

### Role of the funding source

This work was supported by Kavli Trust (grant 38/19). The funder had no role in considering the study design or in the collection, analysis, interpretation of data, writing of the report, or decision to submit the article for publication.

## Results

**Fig. 1:**
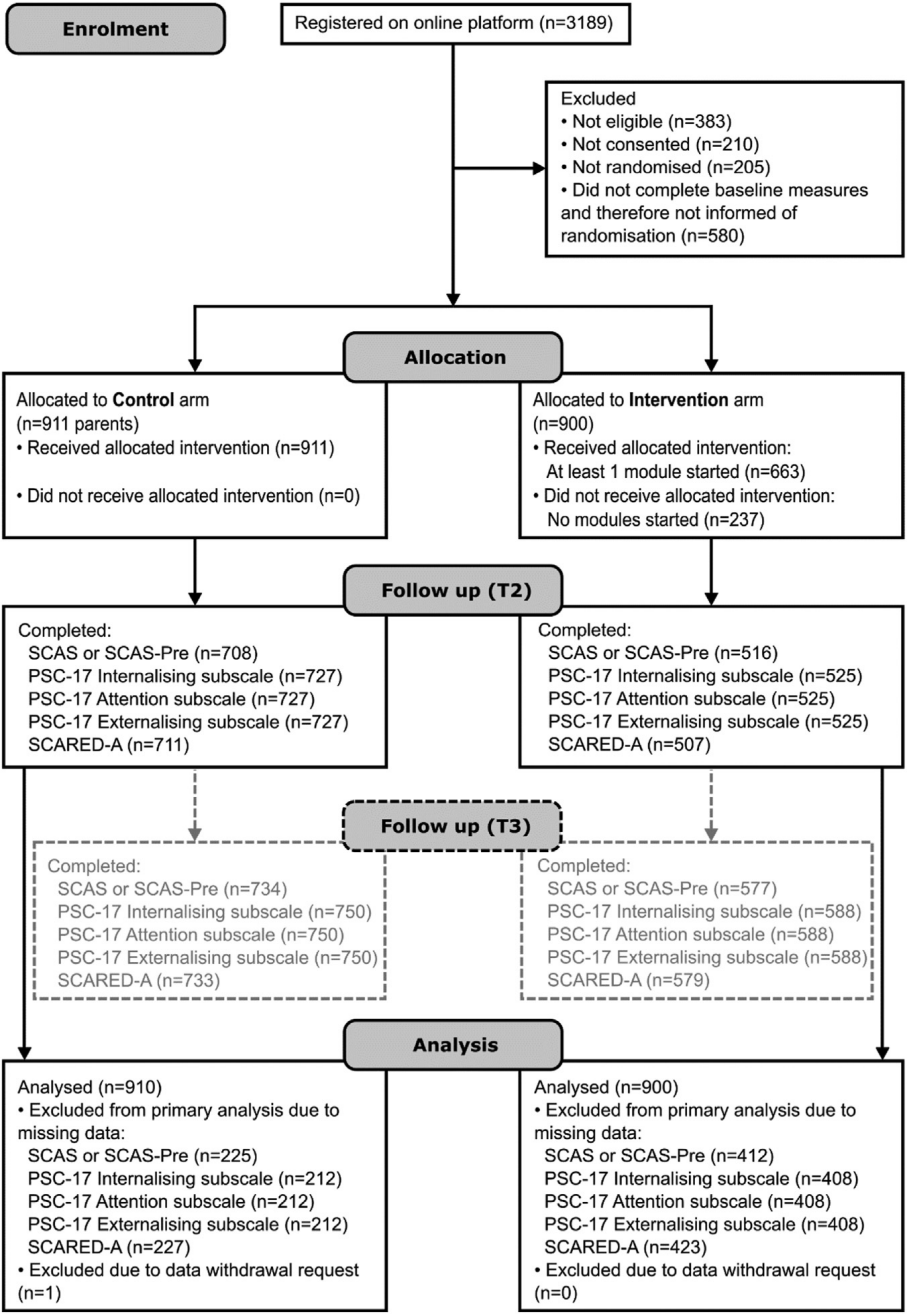
CONSORT flow diagram. T3 is shown in dashed boxes as this time point was not part of the primary analysis.

Between February 15th 2021 and September 30th 2022, 3189 individuals registered with the online trial platform, of whom 1811 (56.8%) participants were assessed as eligible, gave consent, completed all baseline measures and were randomised into the two trial arms (control = 911 and intervention = 900). Of 1811 randomised participants, 71 (3.9%) withdrew during the study (control = 25; intervention = 46) for the following reasons: not having time to continue (control = 8; intervention = 28); preference for alternative trial arm (control = 7; intervention = 0); not having time to complete questionnaires (control = 3; intervention = 5); personal circumstances changed (control = 0; intervention = 3); not enjoying the course (control = 1; intervention = 0); course unhelpful (control = 0; intervention = 1); other/not-specified (control = 6; intervention = 9). Participant retention (based on primary outcome completion) at T2 (6-months post consent) was 67.6% overall (n = 1224 (control = 77.8%, n = 708; intervention = 57.3%, n = 516)) and at T3 (between 08/03/23 and 22/04/23) was 72.4% overall (n = 1311 (control = 80.7%, n = 734; intervention = 64.1%, n = 577)). The trial closed after the target sample size was attained and following a two-week window for recently recruited participants to complete the sign–up processes. See Fig. 1 for CONSORT [Consolidated Standards of Reporting Trials] flow chart.

### Participant characteristics

Analysis was conducted on 1810 participants (control = 910; intervention = 900) using complete case analysis following intention to treat (ITT) principles, with data having been withdrawn by one participant. Baseline demographic characteristics for participants and index children are shown in Tables 2 and 3. Most participants were female (92.7%), with median age of 39 (IQR 35.0–42.0), identified as White British (85.3%) and were financially secure (comfortable: 36.2%; managing: 50.6%; struggling: 13.2%). Parents reported high levels of current anxiety: 96.8% scored at or above the suggested clinical cut-off score on the SCARED-A (≥20 points for males; ≥30 points for females).

**Table 2:** Descriptive statistics of baseline parent demographics by arm and overall.

	Control arm (n = 910)			Intervention arm (n = 900)			Overall (n = 1810)		
	Median	IQR	n	Median	IQR	n	Median	IQR	n
	**n**	**%**		**n**	**%**		**n**	**%**	
**Age**	39.0	35.0–42.0	906	39.0	35.0–42.0	897	39.0	35.0–42.0	1803
**Gender at birth**									
Male	56	6.2		68	7.6		124	6.9	
Female	848	93.2		829	92.1		1677	92.7	
Missing/prefer not to say	6	0.7		3	0.3		9	0.5	
Total	910	100.0		900	100.0		1810	100.0	
**Ethnicity**									
White	808			796			1604		
Mixed	73			82			155		
South Asian	10			8			18		
Other Asian	3			2			5		
African/Caribbean	3			7			10		
Any Other	7			2			9		
**Financial status**									
Comfortable	313	34.7		338	37.7		651	36.2	
Managing	470	52.1		441	49.2		911	50.6	
Struggling	119	13.2		118	13.2		237	13.2	
Total	902	100.0		897	100.0		1799	100.0	
**Treatment for parent anxiety in past 12 months**									
No	370	41.0		378	42.1		748	41.6	
Yes	532	59.0		519	57.9		1051	58.4	
Total	902	100.0		897	100.0		1799	100.0	
**Education**									
Left school before 16	24	2.7		22	2.5		46	2.6	
Left school at 16	66	7.3		62	6.9		128	7.1	
Left school 17/18	50	5.5		52	5.8		102	5.7	
Completed college	175	19.4		147	16.4		322	17.9	
Completed university	587	65.1		614	68.5		1201	66.8	
Total	902	100.0		897	100.0		1799	100.0	
**Number of children**									
1	390	42.9		384	42.7		774	42.8	
2	427	46.9		433	48.1		860	47.5	
3	76	8.4		68	7.6		144	8.0	
4	14	1.5		11	1.2		25	1.4	
5	1	0.1		3	0.3		4	0.2	
6	2	0.2		1	0.1		3	0.2	
Total	910	100.0		900	100.0		1810	100.0	
**Lives with other parent of index child**									
No	165	18.1		156	17.3		321	17.7	
Yes	745	81.9		744	82.7		1489	82.3	
Total	910	100.0		900	100.0		1810	100.0

**Table 3:** Descriptive statistics of baseline index child demographics by arm.

	Control arm (n = 910)			Intervention arm (n = 900)			Overall (n = 1810)		
	Median	IQR	n	Median	IQR	n	Median	IQR	n
**Age**	6.0	4.0–8.0	903	6.0	4.0–8.0	897	6.0	4.0–8.0	1800
	n	%		n	%		n	%	
**Gender**									
Male	466	51.2		456	50.7		922	50.9	
Female	437	48.0		441	49.0		878	48.5	
Missing/prefer not to say	7	0.8		3	0.3		10	0.6	
Total	910	100.0		900	100.0		1810	100.0	
**Ethnicity**									
English/Welsh/Scottish/Northern Irish/British	759	84.0		757	84.4		1516	84.2	
Irish	4	0.4		5	0.6		9	0.5	
Any other White background	45	5.0		34	3.8		79	4.4	
White and Black Caribbean	16	1.8		16	1.8		32	1.8	
White and Black African	11	1.2		8	0.9		19	1.1	
White and Asian	20	2.2		29	3.2		49	2.7	
Any other Mixed/Multiple ethnic background	26	2.9		29	3.2		55	3.1	
Indian	8	0.9		5	0.6		13	0.7	
Pakistani	0	0.0		3	0.3		3	0.2	
Bangladeshi	2	0.2		0	0.0		2	0.1	
Chinese	3	0.3		1	0.1		4	0.2	
Any other Asian background	0	0.0		1	0.1		1	0.1	
African	1	0.1		4	0.4		5	0.3	
Caribbean	2	0.2		1	0.1		3	0.2	
Any other Black/African/Caribbean background	0	0.0		2	0.2		2	0.1	
Arab	2	0.2		1	0.1		3	0.2	
Any other ethnic group	5	0.6		1	0.1		6	0.3	
Total	904	100.0		897	100.0		1801	100.0	
**Developmental disability**									
No	794	87.8		799	89.1		1593	88.5	
Yes	110	12.2		98	10.9		208	11.5	
Total	904	100.0		897	100.0		1801	100.0	
**Treatment for anxiety in past 12 months**									
No	755	90.9		770	93.6		1525	92.2	
Yes	76	9.1		53	6.4		129	7.8	
Total	831	100.0		823	100.0		1654	100.0	

The median age of index children was 6 years (IQR 4.0–8.0), 50.9% were male and the majority were White British (84.2%). Many of the children were reported as having clinically relevant anxiety: 22.8% (obsessive compulsive subscale) to 63.2% (panic and agoraphobia subscale) and 11.5% were reported to have a developmental disability.

### Intervention uptake

Uptake of the intervention was highly variable: 73.7% (n = 663) of participants started at least 1 module, 32.0% (n = 272) completed two or more modules and 19.0% (n = 196) completed the full intervention (8 modules). Individual module completion rate and further engagement data is reported in the Supplementary Materials.

### Intervention efficacy

#### Outcomes at time 2

##### Primary outcome: child anxiety (SCAS-P/Preschool SCAS)

**Fig. 2:**
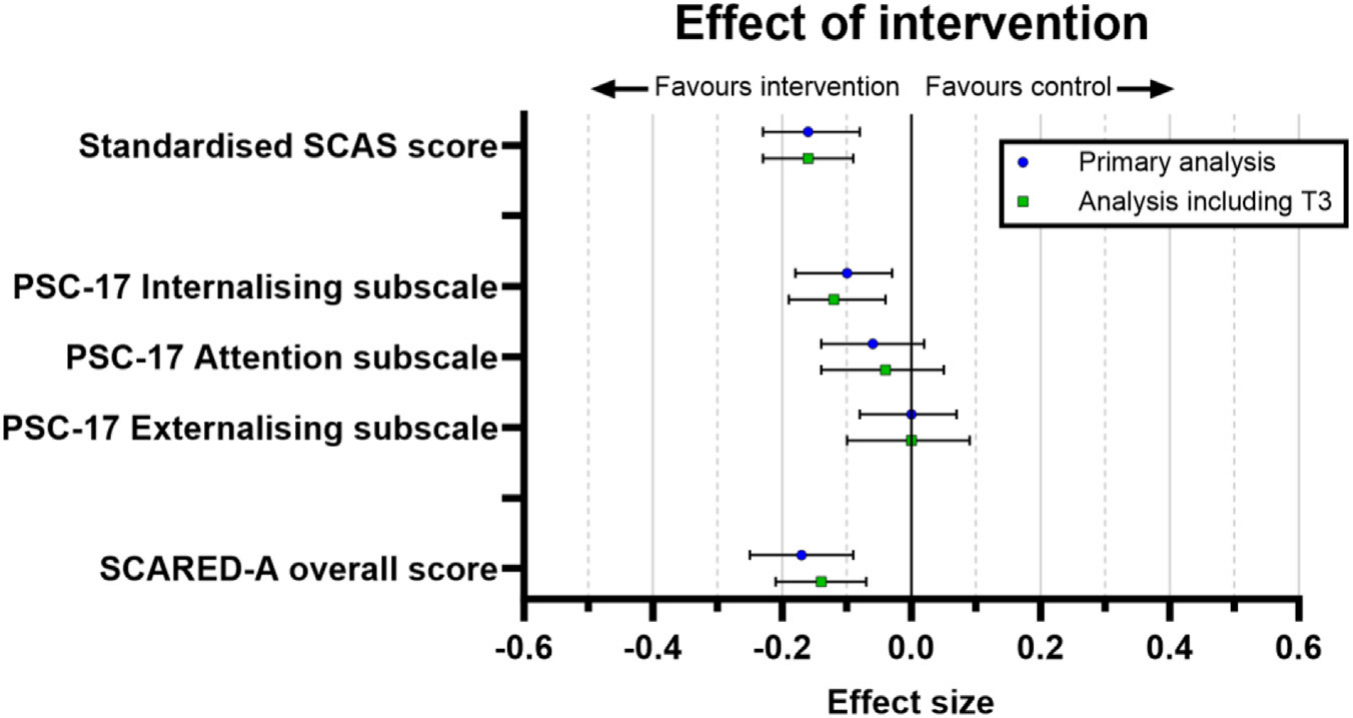
Visualisation of intervention effect sizes for primary outcomes at T2 and T3.

The model included 1173 parents who had completed SCAS-P/Preschool SCAS at T2. A number of participants were excluded from the model for the reasons outlined above. Fig. 2 shows effect sizes for primary and secondary child and parent outcomes.

There was very strong evidence that those in the intervention arm had lowered child anxiety (standardised SCAS score) compared to the control arm, with an effect size (Cohen's d) of −0.16 (95% CI: −0.23 to −0.08, p < 0.001). The difference in standardised SCAS score between the arms was −0.15 standard deviations. On the original scales for SCAS-P (0–114) and Preschool SCAS (0–112), this corresponds to a reduction of −2.38 (95% CI: −3.59 to −1.16) and −2.68 (95% CI: −4.05 to −1.31), respectively.

##### Secondary outcomes

###### Child mental health symptoms (PSC)

The model fitted for the PSC internalising subscale score at T2, adjusting for PSC internalising subscale score at T1, SCAS score at T1, SCARED-A overall score at T1, parent birth gender, index child gender and child age included 1190 parents. There was strong evidence that the intervention was associated with lowered PSC internalising subscale score compared to control, with an effect size of d = −0.10 (95% CI: −0.43 to −0.07, p = 0.007).

An equivalent model fitted for the standardised PSC attention subscale score identified minimal intervention effect at T2 (95% CI: −0.39 to 0.05, p = 0.122, d = −0.06). Similarly, no evidence of an effect for the intervention was found for the PSC externalising subscale score at T2 (95% CI: −0.24 to 0.21, p = 0.910, d = −0.00).

##### Parental anxiety

The model fitted for SCARED-A parental anxiety overall score at T2, adjusting for SCARED-A overall score at T1, SCAS score at T1, parent birth gender, index child gender and child age, included 1160 parents and provided strong evidence that the intervention improved SCARED-A overall score compared to control, effect size d = −0.17 (95% CI: −5.10 to −1.93, p < 0.001).

#### Outcomes at T3

Multiple linear mixed effects regression models were fitted for the primary outcome measures at T3, with a random effect for participant and the fixed effects listed above. Effects were similar to those from the models for T2, suggesting that the intervention effects were maintained longer-term: Standardised SCAS scores compared to control (95% CI: −0.22 to −0.08, p < 0.001, d = −0.15); PSC internalising subscale (95% CI: −0.46 to −0.10, p = 0.002, d = −0.12); and SCARED-A (95% CI: −4.24 to −1.45, p < 0.001, d = −0.14). As expected, there was no evidence that the intervention affected PSC attention scores (95% CI: −0.39 to 0.15, p = 0.384, d = −0.04) and externalising subscale scores (95% CI: −0.30 to 0.28, p = 0.96, d = 0.00). Model ICCs ranged from 0.61 (SCARED-A) to 0.76 (PSC externalising subscale). See Fig. 2 for visualisation of intervention effects at T2 and T3.

### Moderators

The primary outcome model at T2 was refitted to assess moderators of the intervention effect (the full list of moderators and tabulated effects are presented in Supplementary Materials). SCAS-P/Preschool SCAS (child) separation anxiety, social phobia and generalised anxiety subscales were moderators, with greater intervention effects in those with likely clinical levels of symptoms. Similarly, standardised SCAS (child anxiety) overall score at baseline was a moderator: Each standardised SCAS unit increase (1 SD at baseline) was associated with a −0.12 (95% CI: −0.20 to 0.04) units (SDs at T2) increase in the intervention effect (standardised SCAS-P/Preschool SCAS score at T2).

### CACE (intervention engagement) analysis

**Fig. 3:**
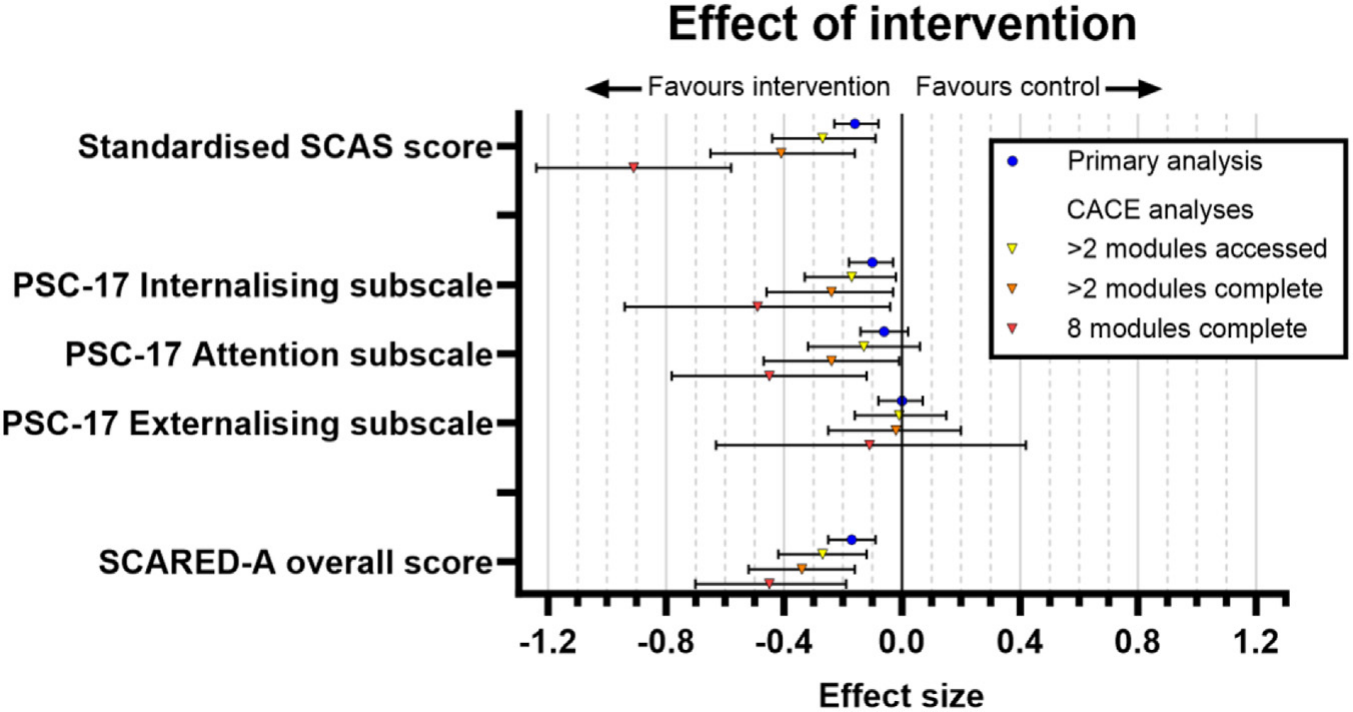
Visualisation of intervention effect sizes for primary outcomes using complete case analysis following intention to treat (ITT) principles and CACE principles, with compliers defined as those beginning >2 modules, completing >2 modules and completing 8 modules.

CACE models suggest that increased engagement with the intervention was associated with increased intervention effects for the primary outcome (SCAS), along with the PSC-17 internalising and attention subscales, and SCARED-A (parent anxiety). The PSC-17 externalising subscale score showed no effect of level of engagement with the intervention. The impact of increased engagement was most apparent for standardised SCAS (child anxiety) scores, where effect size increased from −0.16 to −0.91 under complete case analysis following intention to treat (ITT) principles analysis, when ‘compliance’ was defined as completing all eight modules (see Fig. 3 for visualisation).

### Sensitivity analyses

Sensitivity analyses were carried out to assess the effect of differential attrition under the missing at random (MAR) assumption, and under the assumption that MAR is violated. The primary outcome models were refitted using full information maximum likelihood (FIML, using Structural Equation Modelling (SEM)), and were found to be equivalent to the primary outcome analysis (visualisation of adjusted intervention effects for primary outcomes under complete case analysis and using full information likelihood are contained in Supplementary Materials). The Stata module RCTMISS was also used to assess the effect of different assumptions about the missing data (see Supplementary Materials).

### Harms

No adverse events were recorded during the trial. A subset of parents in both trial arms reported worsening anxiety on SCARED-A between T1 and T2. However, this increase in parental anxiety was more apparent for participants in the control arm than the intervention arm (35.5% v 26.2%, respectively). Responses to the study distress questionnaire indicated a similar magnitude of negative experiences reported by participants in both trial arms (See Supplementary Materials for details).

## Discussion

The intergenerational transmission of anxiety from parent to child is a matter of public health concern. Our study has found that an online intervention targeting highly anxious parents can positively impact anxiety and internalising symptoms in their children, as well as anxiety in the parents themselves, with results stable up to two-years later. This is despite the intervention being unsupported by any clinician input.

These results are a clear signal that an inexpensive, highly scalable, digital intervention can work to prevent the intergenerational transmission of problematic anxiety. Given the prevalence of high parental anxiety, this intervention has the potential for considerable population health impact when administered at scale.

As expected, engagement with the intervention was highly variable. However, the results showed a clear dose–response relationship, with analysis of those who engaged fully (completed all available modules) returning an effect size of −0.91 on the primary outcome (child anxiety) which is comparable to the effect sizes seen for face-to-face interventions for existing child anxiety diagnoses.^30^ Similarly, there was variable engagement in outcome data collection which has been similarly identified in trials of digital perinatal mental health interventions and which is discussed in further detail below.^31^

Unsurprisingly, given the scarcity of interventions targeting the intergenerational transmission of poor mental health, there are no directly equivalent studies for comparative purposes: A 2022 review on preventative interventions for parents with mental illnesses identified only one paper that was not focused on adjuncts to treatment for existing child anxiety and which offered promising results.^1^ Similarly, the face-to-face intervention from which this digital intervention was developed identified a reduction in child anxiety in the intervention group, compared to a small increase in the control group (overall 16% fewer symptoms in the intervention arm at 12-month follow-up). However, this face-to-face intervention is more resource intensive and much less scalable than its digital counterpart.^12^

If we consider digital mental health interventions aimed at treating (rather than preventing) anxiety in children, a recent meta-analysis (7 studies) found them to be highly effective,^2^^,^^19^ with a standardised mean difference and 95% CI of −0.52 [95% CI: −0.90 to 0.14]). However, while useful in providing context, these studies are not comparable to the current study which is preventive in focus and thus had no requirement for the child to be displaying any symptoms of anxiety at baseline. Prevention studies will always be expected to return lower effect sizes than those which attempt to treat existing conditions, because a proportion of participants in prevention trials are healthy and at low risk of developing difficulties. These individuals will experience a ‘floor effect’ whereby no intervention effect will be visible, no matter how powerful the intervention. Moreover, the present intervention was entirely unguided by a clinician, unlike most of the studies in the meta-analyses. This approach, which generates clear cost-savings and much improved scalability, inevitably results in lower effect sizes. Finally, the present intervention sought to effect change in child symptoms indirectly, through the mechanism of parental behaviour, whereas those outlined above attempted to bring about change by intervening directly with the child. Again, although associated with stronger effect sizes, such interventions are considerably more costly and less scalable than the intervention described in the present study.

We can also compare the results of the present study with other efforts towards preventing anxiety in children. In a recent meta-analysis of universal school-based anxiety prevention interventions, an effect size of 0.16–0.26 (post-intervention v medium-term follow up, respectively) was reported.^32^ These results are comparable to those reported for the present intervention yet derive from interventions that were generally delivered face-to-face and are thus more expensive and less scalable.

The digital mental health domain comprises an abundance of unregulated and under-evaluated online interventions and apps.^33^ In contrast, the efficacy of this intervention has been established in an exceptionally large and rigorous trial. Moreover, the intervention itself was adapted from an evidence-based face-to-face group intervention that was delivered in mental health primary care settings (NHSTT).^12^

The results of our trial are particularly noteworthy given that it is an inexpensive, clinically unguided online intervention which has the capacity to be implemented at scale. According to Carey et al., (2023)^34^ apparently small effect sizes “can have large and meaningful impacts, particularly when applied to large populations.” In their modelling, they demonstrated that a small effect size relating to change in scores on the Moods and Feelings questionnaire (MFQ) could result in substantial shifts in the distribution in children's mental health symptoms, such that numbers of those at the rightwards tail of the distribution (those in the clinical range) increased markedly. In their example, a small increase in MFQ score (effect size 0.14) across the population could lead to a 16% increase in CAMHS referrals. The obverse is also true: a small reduction in scores, across a large population, could lead to meaningful reductions of referrals.

However, a clear limitation of the trial is the substantial differential follow-up rates between intervention and control arms (intervention arm = 57.3% (516/900) control = 77.8% (708/910)). This reflects a systematic review of digital perinatal mental health interventions which identified similar levels of differential follow-up data collection between trial arms for web-based interventions but not for digitals apps.^31^ While we cannot know why these rates of follow-up differ, we hypothesise that this reflects a carry-over effect, whereby disengagement from the online intervention (which was common, see above) extended to the trial as a whole (i.e., if a participant ignored ‘nudges’ to re-engage with the online course, they may then have been more likely to ignore emails about data collection). However, our sensitivity analysis concluded that this issue was unlikely to have substantially impacted the results, and an exploration of those who were lost to follow-up suggested that those lost in the two arms did not differ in meaningful ways.

A second limitation of the trial is the relatively homogenous sample. Despite substantial efforts to recruit fathers, the sample is majority female. It is also disproportionately White British, educated, and economically secure. This bias indicates that while digital interventions have the potential to increase equity in access, they may also perpetuate longstanding inequalities present in both mental health research and service provision. The trial team plan to undertake future activities to engage under-represented groups.

A third limitation of the trial is that the trial statisticians conducting the analysis were unblinded during analysis and had access to the outcome data during production of this SAP. While this introduces a risk of bias, it is a common approach within large online clinical trials where the trial statistician is involved in critical decision-making, for example, monitoring data quality. It should be noted that the statisticians conducting the analyses were employed by an independent Clinical Trials Unit.

A fourth limitation is the fact that the intervention completion rates were so variable, with many parents not engaging with the intervention at all. The results suggests that outcomes improved markedly with higher levels of engagement, and efforts should now be directed towards finding means of increasing engagement.

Finally, although the study set out to recruit parents with a range of anxiety severities, including those in the sub-clinical range, in event, almost 97% of participants scored in the likely clinical range on our measures. Therefore, we know less than we would have liked about the utility of this intervention in parents with sub-threshold anxiety. It should also be noted that, owing to the very large sample size, we were unable to carry out diagnostic interviews with participants, so our efforts at determining whether parents were experiencing sub-threshold or fully diagnosable anxiety disorders should only be viewed as estimates.

We believe that the results of this trial are an important milestone in efforts to limit anxiety in a group of children at risk of developing it, namely the children of highly anxious parents. It offers persuasive evidence that a brief, unsupported, online intervention for parents, delivered at minimal cost, can reduce anxiety for both children *and* their parents.

Most adults with anxiety never receive any treatment for their mental health condition. However, 1800 parents with clinically significant levels of anxiety, who were not recruited via health services, chose to engage with this trial. This is highly promising in terms of delivering support to parents and children at risk of anxiety disorders who may be otherwise unengaged with services.

The clear dose–response effect identified in this study indicates that increased parental engagement with the intervention is associated with a greater degree of positive impact on child anxiety. Indeed, for the subset of parents who engaged fully with the intervention, the results were equivalent to those seen in face-to-face interventions for children with existing anxiety disorders.^30^ The extant research into digital interventions indicates that there is substantial potential to increase engagement by embedding the intervention in routine clinical care,^35^ and in such conditions, we can expect the effect size to be substantially larger. As such, this digital intervention could offer a low-cost adjunct to existing support for the high volume of adults with high anxiety who present to primary (mental and physical) health care services and who are parents. However, we should emphasise here that we do not see this as an intervention whose access should be restricted to parents receiving treatment for their own anxiety: it should also be available to the majority of highly anxious parents who will never access mental health services.

To implement this intervention so that it reaches the large population of anxious parents in the UK and beyond requires investigation into two core and currently unanswered questions: First, given the close relationship between level of engagement with the intervention and outcomes, what are the most cost-effective mechanisms for increasing participant engagement, both within and outside healthcare services? Second, what are the barriers and facilitators to the delivery of this intervention within healthcare services, and what approaches will advantageously engage with them? Drawing upon behavioural economics, digital best practice and implementation science, this future research will facilitate the planned wide-spread dissemination of this intervention, with the aim of improving outcomes for children and adults across the UK.

## Contributors

AD: investigation, methodology, project administration, supervision, writing—original draft, and writing—review & editing; JA: data curation, project administration, software, validation, writing-review & editing; AA: investigation, project administration, writing—review & editing; SB: data curation, formal analysis, methodology, writing—review & editing; CEP: conceptualisation, investigation, and writing—review & editing; RE: conceptualisation, methodology, writing—review & editing; CJ: data curation, formal analysis, methodology, software, visualisation, writing—review & editing; PL: conceptualisation, methodology, writing—review & editing; KJL: conceptualisation, methodology, writing—review & editing; NM: conceptualisation, investigation, writing– review & editing; JS: conceptualisation, methodology, writing—review & editing; AT: investigation, writing—review & editing; SCH: conceptualisation, funding acquisition, investigation, methodology, project administration, supervision, validation, writing—review & editing.

## Data sharing statement

Anonymised data is accessible on the Figshare data repository (International registered report identifiers: DERR1-10.2196/40707).

## Declaration of interests

All authors have completed the Unified Competing Interest form (available on request from the corresponding author) and declare no support from any organisation for the submitted work beyond payments received through the grant awarded by the KAVLI Trust. PL has grant funding from Wellcome Trust and Economic and Social Research Council, all other authors have no financial relationships with any organisations that might have an interest in the submitted work in the previous three years. SB is participant on the following Boards (ACER (NIHR130693); NEON (NIHR RP-PG-0615-20016); ODDESSI (NIHR RP-PG-0615-2002)) all other authors have no other relationships or activities that could appear to have influenced the submitted work. SC-H designed the digital intervention which this trial evaluated, and funded its development.
