# Supplementary material for: Effectiveness of an unguided modular online intervention for highly anxious parents in preventing anxiety in their children: a parallel group randomised controlled trial
Source: Lancet Reg Health Eur. 2024 Sep 4;45:101038. doi: 10.1016/j.lanepe.2024.101038 (PMC11405817; doi:10.1016/j.lanepe.2024.101038)
Supplement: Protocol.docx [file mmc2.docx]

**PROTOCOL**

***Full Project title****:* A Randomised Controlled Trial of an Online Intervention to Prevent Anxiety in the Children of Anxious Parents

***Short Title:*** Parenting with Anxiety: Helping anxious parents raise confident children

***Research Reference Number****:* ER/SC430/1

***Protocol Version Number and Date****:* v6.1 6th February 2023

***Name of PI***: Prof Sam Cartwright-Hatton.

***Host institution***: University of Sussex, UK.

***Funder***: Kavli Trust

# Table of Contents

[Table of Contents 1](#_Toc103674855)

[Study Summary 3](#_Toc103674856)

[1. Rationale and background information 5](#_Toc103674857)

[2. Study goals and objectives 8](#_Toc103674858)

[2.1. Goals 8](#_Toc103674859)

[2.2. Primary objective 8](#_Toc103674860)

[2.3. Secondary objectives 8](#_Toc103674861)

[3. Study Design 8](#_Toc103674862)

[3.1. Study setting 9](#_Toc103674863)

[3.2. Participant Eligibility Criteria 9](#_Toc103674864)

[4. Methodology and Participant Timeline 10](#_Toc103674865)

[5. Intervention 12](#_Toc103674866)

[5.1. Changes to the intervention 14](#_Toc103674867)

[6. Recruitment 14](#_Toc103674868)

[6.1. Participant identification 14](#_Toc103674869)

[6.2. Screening 16](#_Toc103674870)

[6.3. Payment 16](#_Toc103674871)

[6.4. Consent 16](#_Toc103674872)

[7. Randomisation scheme 16](#_Toc103674873)

[8. Blinding/unmasking 17](#_Toc103674874)

[9. Data collection 17](#_Toc103674875)

[10. Study Within a Study (SWAT) 20](#_Toc103674876)

[11. Statistics and data analysis 21](#_Toc103674877)

[11.1. Sample size 21](#_Toc103674878)

[11.2. Planned recruitment rate 21](#_Toc103674879)

[11.3. Statistical analytical plan 22](#_Toc103674880)

[11.3.1. Primary and secondary outcome analysis 22](#_Toc103674881)

[11.3.2. Interim analysis 23](#_Toc103674882)

[11.4. Procedure to account for missing or spurious data 23](#_Toc103674883)

[12. Data Management, Protection & Confidentiality 24](#_Toc103674884)

[13. Ethical and regulatory considerations 25](#_Toc103674885)

[14. Participant Safety 26](#_Toc103674886)

[14.1. Research ethics committee and reports 27](#_Toc103674887)

[14.2. Peer review 27](#_Toc103674888)

[14.3. Public and Patient involvement 28](#_Toc103674889)

[14.4. Post trial care 28](#_Toc103674890)

[15. Dissemination policy 28](#_Toc103674891)

[16. Expected Outcomes of the Study 29](#_Toc103674892)

[17. Description of the research group(s)/research environment(s) 30](#_Toc103674893)

[18. Leadership 31](#_Toc103674894)

[19. Funding and support 31](#_Toc103674895)

[References 32](#_Toc103674896)

[Appendices 35](#_Toc103674897)

[Table 1: Intervention Modules and Content 13](#_Toc67997085)

[Figure 1: Participant Flow Diagram 11](#_Toc67997086)

# Study Summary

| Study Title | A Randomised Controlled Trial of an Online Intervention to Prevent Anxiety in the Children of Anxious Parents |
| --- | --- |
| Short Title | Parenting with Anxiety: Helping anxious parents raise confident children |
| Study Design | A randomised controlled trial of an intervention to reduce symptoms of anxiety in the children of anxious parents. Parents will participate in an online intervention which helps them develop a calm, consistent, behaviour management style. The parents will be randomised to the intervention or a control group with no intervention. The intervention itself will undergo a component analysis to determine whether some modules are more effective than others. |
| Study Participants | 1754 parents, aged 16+, with a child aged 2 to 11 years (inclusive), with significant self-reported levels of current or lifetime anxiety will be randomised into the study. |
| Follow up duration | Participants will be followed up at 6 months and depending on when they were recruited, some will also be followed up between 9-25 Months. |
| Planned Study Period | 40 Months Total. This includes 9 months set up. 22 months recruitment, 6 months to complete follow up. 3 months data analysis and dissemination. |
| Research Question/Aim(s) | This study will test the effectiveness of an online intervention that aims to prevent anxiety in the children of anxious parents, and provide information for the optimisation of this intervention. |

**FUNDING AND SUPPORT IN KIND**

**FUNDER(S)**

This study has been funded by the Kavli Trust. The study is also supported by the University of Sussex who have waived all overheads**.**

**ROLE OF STUDY SPONSOR AND FUNDER**

The University of Sussex will be sponsoring this study, and will assume overall responsibility for the initiation and management of the study. The Sponsor may delegate some duties to members of the research team and to the Brighton & Sussex Clinical Trials Unit, who will be managing the conduct of this study. These duties will be laid out clearly in a Delegation of Sponsor Duties document.

**ROLES AND RESPONSIBILITIES OF STUDY MANAGEMENT COMMITEES/GROUPS & INDIVIDUALS**

- **Trial Management Group**

The TMG will consist of all members of the research team, including co-applicants, user representatives and members of the Brighton and Sussex Clinical Trials Unit. They are planning to meet monthly to discuss study progress.

- **Trial Steering Committee**

The TSC will consist of a minimum of three Independent members (a statistician, and two clinicians of relevant fields) alongside members of the research team and two PPI members. Their role would be to provide overall supervision for the trial on behalf of the Sponsor and the Funder, to ensure the trial is conducted according to the Research Governance Framework for Health and Social Care and all relevant regulations and policies.

# Rationale and background information

Anxiety disorders are the most common psychiatric condition of pre-adolescence (**Cartwright-Hatton**, McNicol, & Doubleday, 2006). Moreover, anxiety runs in families: meta-analysis shows that children of anxious parents are twice as likely to have anxiety problems than children of the non-anxious (**Lawrence**, Murayama, & Creswell, 2019). This intergenerational transmission of anxiety largely relies on environmental rather than genetic processes (Eley et al., 2015); research from our team shows a number of behaviours in anxious parents that are anxiogenic to children (e.g., **Cartwright-Hatton**, Abeles, Dixon, Holliday, & Hills, 2013; Field et al., 2017; **Lester**, Field, & **Cartwright-Hatton**, 2012). This problem affects children across the world, regardless of gender, ethnicity, socioeconomic status (SES) or location.

We have developed a brief, group-based intervention to prevent this transmission of anxiety: It helps parents develop a calm, consistent behaviour management style, whilst learning skills to discourage children’s avoidance. It helps parents to identify areas where anxiety might impact on their parenting, and make plans to minimise this. A UK National Health Service-based RCT of the face-to-face version resulted in 16% fewer anxiety symptoms in participants’ children, compared to controls (**Cartwright-Hatton** et al., 2018). But, the bulk of anxious people never reach services: In the UK, primary care services aim (though often fail) to reach 20% of clinically anxious people. The children of the 80%+ who do not access services are likely to be particularly vulnerable. Moreover, in our published RCT, and in our clinic that offers this intervention, around half of interested parents fail to attend because of childcare/work commitments or because of anxiety about the group-setting. To reach these under-serviced families, we will conduct an RCT of an *online* version of the intervention. We will recruit a very large sample of anxious parents, and examine impact on child and parent emotional outcomes.

Fathers are particularly disadvantaged by these barriers to access, and represent only 15% of parents attending our clinic. Indeed, interventions targeted at parents are overwhelmingly focused on mothers: In a systematic review of fathers’ inclusion in parenting interventions, Panter-Brick et al. (2014) reported that the vast majority of studies included few or no fathers, and only a handful disaggregated data by parent gender. There are many reasons for this neglect of fathers: In particular, is an assumption (from families and professionals) that attending to children’s health and emotional needs is the mother’s role. As a result, interventions tend to focus on recruiting mothers, and provide interventions that suit them (e.g., in office hours, which discriminates against fathers). This is problematic: There is clear evidence that fathers have a key role to play in children’s emotional development (e.g. Barker, Iles, & Ramchandani, 2017; Ramchandani & Psychogiou, 2009). Moreover, it is possible that fathers who experience significant anxiety might have different and potentially *more deleterious* impacts on child outcomes than anxious mothers (e.g., Bögels & Perotti, 2011; Bögels & Phares, 2008). Therefore, as recommended by Panter-Brick et al. (2014) we will target extra recruitment efforts at fathers and deliver the intervention online (which is likely to remove some barriers for fathers) and disaggregate our data by parent gender when reporting.

Prevention of anxiety is in its infancy, particularly prevention targeted via anxious parents. Whilst we have good models of the processes involved in the intergenerational transmission of anxiety, we know little about what is necessary and sufficient for prevention to be effective. So, our intervention is currently broad-based, covering a wide range of components that could be important (see ‘Intervention’ below). In order to maximise efficiency for parents (and services) we need to understand which components are most effective and for whom, particularly in online format and for the wider, more inclusive group that is likely to access this. To achieve this the current study will evaluate the effectiveness of the intervention in online form, with a broad range of anxious parents. Our uniquely large sample will allow us to conduct a component analysis of our intervention. In doing so, we will not only optimise the intervention, but gain a better theoretical understanding of the mechanisms underpinning intergenerational transmission of anxiety.

We believe that there is value in increasing confidence in a child who is only at risk of sub-threshold anxiety, and in reducing severity of symptoms in a child with an ongoing anxiety disorder. Therefore, we take a ‘public health’ approach; i.e., the intervention is intended not just to reduce risk for full-blown diagnosable anxiety disorders, but to reduce symptoms across the anxiety spectrum. Moreover, we do not believe that the intervention is only of benefit to children whose parents have clinically diagnosable anxiety disorders. We will invite any parent who thinks that their level of anxiety could have a negative impact on their child to participate, without requiring a formal anxiety diagnosis. By sampling into the sub-clinical range of parent anxiety, we will maximize preventative opportunities. This may be of particular relevance given the heightened level of anxiety experienced by many parents during and after the COVID-19 pandemic.

To investigate and control for any biases in parent reports of child anxiety, all study participants will be invited to nominate a co-respondent who provides substantial care to the child (e.g. grandparent, co-parent, friend) without being in a financial arrangement with the participant. This co-respondent will be asked to complete scales including a measure of their own anxiety and one on the child’s anxiety at all data-collection points. This will enable us to examine agreement between parent and co-respondent ratings of child anxiety. When a co-parent (but not any other type of co-respondent) is referred they will furthermore complete a parenting behavior scale. This will allow us to examine the impact of this second parent’s anxiety and parenting behaviour on child outcomes. This method of triangulating data is contingent on the participants and co-respondents being willing to engage with this strand of the study.

This study employs an online RCT. These are less burdensome for participants, less costly for funders, and are becoming widespread (Bailey et al., 2013). They widen participation to under-represented groups, e.g. men, which is pertinent to the present study. However, compared to in-person RCTs, study attrition can be high within online studies. We attempt to minimize this though the intervention and study design which has been developed to minimise barriers to engagement by way of accessibility and usability. To promote longitudinal engagement, completion of post-intervention measures will be incentivised by means of a monetary voucher.

# Study goals and objectives

## Goals

This study will test the effectiveness of an online intervention that aims to prevent anxiety in the children of anxious parents and provide information for the optimisation of this intervention.

## Primary objective

**1a**: To investigate the effectiveness of an online, parent-focused intervention for the prevention of anxiety in children of anxious parents. We hypothesise that children and parents in the intervention arm will show significantly fewer anxiety symptoms, (and greater parental wellbeing) at 6-month follow-up, compared to those in the control arm.

## Secondary objectives

**1b**: To determine which components of the intervention have most/least impact on outcomes, and test whether the effect of each component is moderated by participant characteristics (type/severity of parent/child symptoms; SES; child age). In particular, we will test the moderating impact of the gender of the anxious parent on all outcomes.

**1c**: To use our uniquely large, ‘accelerated longitudinal’ dataset (Galbraith, S., Bowden, J., & Mander, 2017) to conduct a prospective exploration of the intergenerational transmission of anxiety. We will focus on parenting behaviour, and the moderating effects of parent/child gender, and child age.

**1d**: To explore the impact of co-parent anxiety severity and parenting behaviours on child outcomes. We hypothesise that child outcomes will be less good where the co-parent is also anxious and/or engages in frequent anxiogenic parenting behaviours.

# Study Design

The population will be anxious male and female adults (aged 16+) who have children aged 2 to 11 years. The study will last for three years.

**Obj 1a:** We will employ a parallel groups RCT design with 2 equally-sized arms (intervention/control) to test the impact of the intervention on parent and child outcomes.

**Obj 1b:** Using a ‘dismantling’ design, we will investigate which elements of the intervention have most impact, and the factors that moderate this. Participants in the Intervention arm will be randomly allocated to one of eight slightly different forms of the intervention. Each of these versions will have one of the modules disabled (but *not* the ‘starter’ module). At baseline, and at 6-months, parents will complete measures assessing anxiogenic behaviours that are targeted in each module. In this way, we will be able to explore the effect of each individual module.

**Obj 2:** We will conduct a novel cross-sectional and prospective investigation of the processes involved in intergenerational transmission of anxiety.

**Obj 3:** We will examine the impact of co-parent anxiety and parenting behaviours on child anxiety using concurrent and prospective data from a sub-set of co-parents.

## Study setting

The study will be completed entirely online, with UK-based, self-referred participants.

## Participant Eligibility Criteria

We have designed eligibility criteria to resemble those that would likely be employed in any eventual roll-out of the online intervention. As such, they are minimal. Participants need to:

- Be a parent (any gender, adoptive/biological/step/foster/grandparent) aged 16+, of a child aged 2 to 11 years (inclusive). The index parent must have at least 50 days’ contact with the index child per year and confirm that they see enough of the child to report on the child’s current anxiety level.
- Index parent must be a UK resident.
- Self-report subjectively substantial levels of current or lifetime anxiety.
- Able to commit to completion of measures at (up to) three time points even if allocated to the control arm.

We will not exclude participants on the basis of current/previous psychiatric treatment (parent or child) or on any psychological, neuropsychological or physical condition.

To ensure that the nominated co-respondents are suitable to participate in the study, we have included brief eligibility criteria. They need to:

- Know the child well enough to answer a questionnaire about their feelings and behaviours.
- Be aged over 16.

# Methodology and Participant Timeline

The entire study will be completed online. Participants will flow through the study as follows:

1. Receive summary information.
2. Be screened against inclusion/exclusion criteria (see ‘eligibility’ above). Those meeting criteria will go to Step iii.
3. Receive information about the study and give consent online.
4. Refer a co-respondent to complete measures. Participants will be asked to provide details of a co-respondent. This person will be emailed from within the study platform 48 hours after the participant has completed measures. A participant can also choose not to refer or to make a referral later.
5. Complete baseline measures.
6. Participant receives email confirming their choice with regard to making a referral (point iv) containing instructions how to change their referral/make a referral.
7. Be randomised. Participant will be informed of the arm of the trial they have been allocated to.
8. Take part in Intervention or Control group
9. Complete outcome measures at 6 months (same as baseline assessment).
10. Depending on when the participants sign up for the study, some will be invited back tocomplete outcome measures again, once, towards the end of the study to allow exploration of longer-term outcomes. This will be administered four-months prior to the end of the study (between 9-25 months post randomisation). This follow-up invitation will be issued to all participants excluding those who submitted their 6-month follow-up questionnaire two months of less prior to the point of invitation.


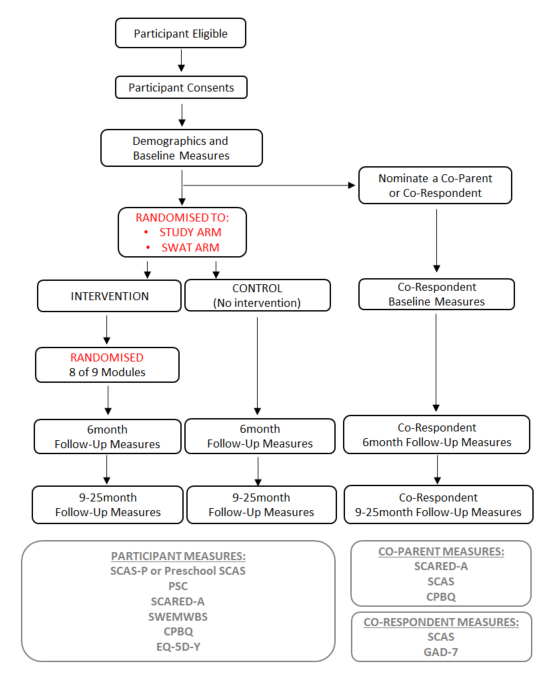


Figure 1: Participant Flow Diagram

During the study, parents will be invited to take part in the following sub-study:

- (**Objective 3**) Participants will be invited to nominate a co-respondent to complete questionnaires. If the co-respondent is a co-parent they will be assessed on their own anxiety, their parenting behaviors and the anxiety of the index child (SCARED-A, CPBQ and SCAS, see below). If the co-respondent is someone other than a co-parent (e.g. grandparent) they will be assessed on their own anxiety and the anxiety of the index child (GAD-7 and SCAS). The co-respondent will complete these measures online, soon after the index parent completes baseline assessment and will also be invited to complete the same measures again at subsequent follow-up points. Co-respondents who completed measures less than two months prior to the point of T3 invitation (01 March 20230 will be excluded from this round of follow-up data collection.

# Intervention

The intervention will be delivered fully online and follows closely the format of the evidence-based in-person version. It has one ‘Starter’ module and eight ‘further’ modules and is completed by parents (children do not participate). The Starter module is completed by all parents. Subsequently, parents complete 7 of 8 further modules (one disabled at random). It is expected that each module will take between 20 and 30 minutes to compete. Each module has accompanying home practice tasks which the participant is encouraged to try out before progressing to the next module.

The emphasis throughout all modules is non-blaming and non-stigmatising. Parents are reassured that there is no such thing as a perfect parent; that all parents have ‘hotspots’ and that the best parents simply know where these are and try to ‘rub the corners’ off them. See Table 1. for overview of module content (further detail contained in appendices)

| **Topic** | **Content** |
| --- | --- |
|  |  |
| Core Topic (Starter) | **All about anxiety and confidence in children**   - General intro - Cognition - Fight-flight |
| Topic A | **The role of avoidance and small steps to reducing it**   - Avoidance mechanism - Fear hierarchy |
| Topic B | **Using play to develop children’s confidence**   - Adventurous play – outdoor, unsupervised - Rough and Tumble - Play with parents |
| Topic C | **Using ‘Emotion Coaching’ with children**   - Noticing - Tuning in to emotions - Managing difficult behaviour using Emotional Coaching Approach |
| Topic D | **Managing difficult behaviour: praise and reward**   - Praise - Rewards - Star Charts |
| Topic E | **The role of sleep, exercise and diet in children’s mental health** |
| Topic F | **Parenting Hotspot: Reducing Overprotection**   - Risks associated with overprotection - Techniques to minimise overprotective behaviour |
| Topic G | **Modelling confident behaviour: compensating for parenting gaps**   - Modelling - Compensation - Mind the Gap |
| Topic H | **Managing difficult behaviour: consequences and limit setting**   - Limit Setting - Consequences - Time out |

Table 1: Intervention Modules and Content

The order in which allocated modules will be displayed to participants will be randomised. However, maximising retention is a priority so participants will be able to select the order in which they complete the modules and they may complete the intervention at their own pace. They will be encouraged to start as soon as possible after randomization, ideally leaving a few days between modules. We will issue three 'nudge’ emails and SMSs to participants who have been randomised to receive the intervention but have not signed in to access the course. These will be issued at the following time points: 3 days, 7 days and 14 days after the initial invitation was sent.

Once a participant has accessed the intervention, they will be invited to share a copy of the intervention (containing their randomised array of modules) with a supporter (e.g.co-parent, friend, relative). The purpose of this is to support the participant in sharing their learning. This mirrored version will not contain any of the participant’s data.

## Changes to the intervention

Given the online nature of the intervention, we retain the option of making alterations to the app in response to certain scenarios:

- External or platform-specific activities which require the application to be updated. This could include resolving issues arising in response to a change in mobile operating systems.
- We may make changes to the content or user interface of the app should a disproportionate level of attrition be evident in one or more modules. This would be evident from the interim analysis (Section 7.3.2). In doing so we would ensure the core content (as benchmarked against the face-to-face intervention) was retained. This will be subject to scrutiny and approval by the Trial Steering Committee.

# Recruitment

## Participant identification

This study is intended to reach anxious parents, the majority of whom never receive treatment for anxiety (although those who have will not be excluded). So, we recruit chiefly from non-NHS sources:

**Recruitment from the Genetic Links to Anxiety and Depression Study (GLAD**) We will make a formal request to recruit from the Genetic Links to Anxiety and Depression Study (GLAD) which is part of the NIHR Mental Health Bioresource. GLAD is the largest ever study into anxiety and depression and has currently recruited 16,000 individuals with anxiety disorders (expected to be approximately 26,000 by commencement of our recruitment). Projects adopted to recruit from GLAD via the Bioresource have access to this large pool of potential participants.

**Mental Health Charities**: We have agreement to recruit from Anxiety UK, which is the UK’s largest anxiety charity, run by and for sufferers. Anxiety UK ran the pilot of the face-to-face intervention. They have over 100,000 social media followers and 2 million website views per annum and Sam Cartwright-Hatton is a patron. We have links with Mind (currently part-fund and host of our in-person intervention) and local charities.

**Male Mental Health Organisations and Organisations Supporting Fathers:** We will establish relationships with relevant organizations to actively target fathers experiencing anxiety.

**Social Media**: We will actively recruit via Facebook, Twitter and other appropriate channels.

**Other**:

We will advertise the study in **health settings** but will not actively ‘recruit’ via clinician referral.

We will leverage the professional networks of the trial management group to bring the study to the attention of relevant organizations and institutions, for example schools, businesses and professional bodies. This may take the form of an organization contacting parents directly e.g. via a school newsletter or staff intranet page. Within the education sector we use relationships such as within Local Authorities and Academy Trusts to advertise the study to teachers and SENCOs who can then choose to share information about the study with parents.

The information given to organizations to share will take the form of REC approved materials which are already in the public domain.

To secure recruitment for this large study, we have costed the services of a public relations company, and a videographer to produce a recruitment video. As well as maximizing recruitment from the above sources, this will enable better exposure via mainstream media (TV, newspapers, radio), and facilitate maximum recruitment, particularly of fathers.

## Screening

Screening will take place online. Participants will respond to eligibility questions. Participants who are not eligible will be informed immediately. Participants who are eligible will proceed to informed consent. Prior to screening, participants will have provided their email address to discourage ineligible participants from making further attempts to access the study.

## Payment

Participants will be paid £15 on completion of 6-month follow-up outcomes and £15 on completion of 9-25-month outcomes, if applicable.

Co-respondents will be paid £10 on completion of baseline outcome measures and £10 on completion of 6-month and 9-25-month follow-up outcomes.

A randomised 50% of index participants will be paid a further £10 if their nominated co-respondent completes baseline measures. This will allow us to investigate whether providing monetary incentives to participants has an effect on the completion rates of referred co-respondents, using a ‘study within a trial’ (SWAT) design (see section 10). All payments will take the form of Amazon vouchers distributed via email.

## Consent

Participants will receive study information and give informed consent online. Participants will be made aware within the online Participant Information Sheet that participation in the study is voluntary. They will also receive a full copy of the Participant Information Sheet and Consent by email.

# Randomisation scheme

We will employ block randomisation, in large blocks, to one of two arms (Intervention or Control (no Intervention)). This will be carried out using predefined lists. Those assigned to the ‘Intervention’ group will be further randomised to one of 8 conditions to allow component analysis (Objective 1b). If a parent has more than one child within the target age-range (2-11) the system will allocate one child at random to report on when responding to questionnaires. Given the large sample size, stratified randomisation is not deemed necessary.

# Blinding/unmasking

As all outcomes will be self-reported by parents, no assessors need blinding. No data entry will be required of the team, so this needs no blinding. As the intervention is delivered digitally and unguided, there are no clinicians to mask. Parents will know whether they are in the intervention or control condition, but not to which of the eight intervention arms they have been assigned. This is a low-risk psychoeducational intervention, so emergency unmasking procedures are thought unnecessary. If unmasking is requested, this will be managed by the steering committee on a case-by-case basis.

# Data collection

Unless otherwise indicated, ‘outcomes’ are those reported by the index parent, in both arms, at baseline, at 6 month and subsequent follow-up. All outcomes are completed online, by the participants. Regular checks will be completed by the Trial Manager to ensure that processes for capturing and storing data are functioning well.

As in our previous research (Cartwright-Hatton et al., 2018) parents will complete measures for just one child and where more than one child is eligible, one child will be selected at random (by computer). Our previous RCTs and feasibility research (Cartwright-Hatton et al., 2018; Cartwright-Hatton et al., 2011) showed low response rate and weak reliability for measures completed by children in this young age group, and by teachers, so these will not be used. For each of the study objectives, we will capture the following outcomes:

**Objective 1a**: *Primary outcome*: Child anxiety. *Secondary outcomes*: Parent anxiety, Parent wellbeing.

**Objective 1b**: Parenting behaviour, as appropriate to each module.

**Objective 2**: A range of variables relating to parenting behaviours and parental anxiety. These will be drawn from measures captured for other objectives.

**Objective 3**: Baseline co-parent behaviour and anxiety.

Below is an overview of assessments. The measures are contained within appendices.

***Child anxiety symptoms***: Spence Children’s Anxiety Scale *(SCAS-P* & *Preschool SCAS)* (Spence, 1997; Spence, Rapee, McDonald, & Ingram, 2001). These parallel instruments are acceptable to parents, and have good validity/reliability. Diagnoses will not be used, as we are taking a ‘public health’ approach; i.e., the intervention is designed to prevent/reduce symptoms across the anxiety spectrum, rather than focusing narrowly on preventing full-blown diagnosable disorders.

***Parents’ and co-parents’ anxiety:*** SCARED-A – an adult version of the SCARED (van Steensel & Bögels, 2014) assessing each of the DSM anxiety disorders, using 71 items. It has good internal consistency and is significantly correlated with results from the ADIS-IV-L diagnostic interview schedule.

***Parents’ wellbeing***: Rather than limit our focus to negative features of parents’ mental health, we will also examine positive features/wellbeing using The Short Warwick Edinburgh Mental Wellbeing Scale (SWEMWBS) (Stewart-Brown et al., 2009). A 7 item self-report measure (rated 1 to 5), of features of positive mental health (positive affect, interpersonal relationships, positive functioning). It has highly correlated with the original 14-item scale (WEMWBS, Tenant et al., 2007) and has high internal consistency and good validity.

***Child emotional and behavioral symptoms:*** The Pediatric Symptom Checklist: PSC-17 (Gardner et al., 1999) is a general mental health screening tool for children. The parent reports how often their child demonstrates internalizing, externalizing or attentional symptoms using three levels of frequency. The scale has good validity and sensitivity with comparable case detection to semi-structured clinical interviews and is widely used as a screen in primary care.

**Child’s health:** EQ-5D-Y Proxy Report (Wille, et al., 2010) is a proxy report measure of health-related quality of life developed for use with children. The parent reports how they would measure the child’s health across five dimensions: mobility, looking after themself, doing usual activities, having pain or discomfort, feeling worried, sad or unhappy, using three levels of severity. It is a version of the EQ-5D-3L, which is widely used in cost-effectiveness analysis and has good validity and internal consistency.

***Study Attrition Rate***: Proportion of participants that complete 6-month follow up measures.

**Study Completion Rate:** Proportion of participants in the intervention arm that complete 3 modules.

**Module satisfaction:** Brief instrument designed to capture participants satisfaction with completed modules. Option to include free-text comment.

***Demographics***: Age, gender, SES, ethnicity, co-parenting status, child age, child developmental disability.

***Adverse events***: Short instrument designed for the study (see ‘ethics & regulatory considerations’ below). Number of children and parents, in each arm, whose anxiety deteriorates.

***Mediators***: We will measure parenting behaviour by asking parents (both arms) to complete measures of parenting behaviours addressed by each module. For most modules, items are taken from the CPBQ (Majdandžić, de Vente, & Bögels, 2016; Majdandžić et al., 2018), a psychometrically strong, self-report instrument measuring parenting behaviours associated with risk of child anxiety. To minimise burden on participants we will employ a psychometrically valid shortened version of the CPBQ, developed for the Parenting with Anxiety study by the scale authors. For the few areas in our intervention not covered by the CPBQ (e.g. sleep, exercise, diet), items have been identified from existing validated instruments or, in some cases, constructed for the purposes of this study.

***Moderators***: Baseline (and, where appropriate, change in): severity/type of parent and child anxiety (SCAS and SCARED-A); SES; Previous parent or child treatment for anxiety; parent and child gender, child age, child developmental disabilities

***Co-respondent data collection:***

If the nominated co-respondent is a co-parent they will complete the SCARED-A, the SCAS and the CPBQ.

If the nominated co-respondent is someone other than a co-parent they will complete the SCAS and the Generalised Anxiety Disorder Assessment (GAD-7) (Spitzer, et al., 2006) a seven-item scale which screens for anxiety disorder.

Maximizing response rate and retention of participants is a priority. So, participants and co-respondents will be offered vouchers for completion of measures. Furthermore, we will employ some of the following evidence-based approaches (Brueton et al., 2014; Edwards et al., 2010) such as: keep questionnaires to a minimum; send pre-notification emails/SMS in advance of data request; send personalized emails/SMS with simple header to request data; begin with most relevant/easiest questions; remind participants of confidentiality; give deadline for response; tell participants others have completed already.

When requesting follow-up data we use a stepped approach. For participants aged less than 18 at the time of contact only steps 1-4 will be utilised.

1. Pre-notification email and SMS
2. Initial email and text contact (automated and personalised in line with principles outlined above)
3. Follow-up email and SMS message (as above)
4. Follow-up email and SMS message
5. Telephone call. The procedure for contacting the participant by phone is as follows:
6. Telephone call to participant. If participant answers, researcher speaks to them using approved script. End of contact.
7. If participant fails to answer, but voicemail is available, researcher leaves a message using approved script. End of contact.
8. If participant fails to answer, but voicemail is NOT available, researcher can make one further attempt at a call (following steps I and II above).

There will be a one-week pause between each of the steps outlined above. Participants will have explicitly consented to being contacted using these methods.

If a response has not been received from a co-respondent one week after the parent or the study team has made the initial contact with them, or, if the co-respondent signs-up but not begin the scales, or, if the co-respondent begin the scales but not complete, the study team will email the co-respondent. If no response within one week, we will email the parent asking them to nominate another co-respondent. Each referring parent will get just this **one** additional invitation to nominate a new co-respondent.

# Study Within a Study (SWAT)

In order to compare the response rates of co-respondents nominated by participants who have and have not been offered a monetary incentive, we will randomize all participants into additional arms where they will receive/not receive a £10 voucher to refer a co-respondent for objective 3. If in the relevant arm, the voucher will be received by the participant after the co-respondent has completed their baseline assessment measures. All participants will be told that the co-respondent will later receive a £10 voucher for completion. Co-respondents will also be invited to complete measures at the 6-month time point and, if applicable, 9-25 months post randomisation. The proportion of co-respondents who complete baseline and 6-month follow-up measures will be compared across SWAT arms.

Full details of the SWAT are contained within the separate study protocol [insert link]

# Statistics and data analysis

## Sample size

The sample size has been calculated to provide adequate power for our key objective (Objective 1a.).

**Objective 1a**: Based on our existing research, for our primary analysis, we anticipate a small effect size (0.2). With 90% power for 5% significance this requires 526 in each of two arms. Allowing 40% attrition (typical for online psychotherapeutic studies) we need to randomise 877 to each arm. **Total = 1754.**

## Planned recruitment rate

Recruitment will be in two stages: a ‘soft’ launch which will run for a period of one month, followed by 22 months of active recruitment. It is anticipated that 85% of the target sample (n = 1490) will be recruited within the first 12 months.

Should the recruitment target be met earlier than the planned recruitment end date (October 2022) we will continue to accept participants into the study. If there is budgetary allowance, these participants will follow the standard participant flow (as outlined in section 4.)

Should budgetary constraints prevent payment for completion of measures, these participants will be invited to join the main trial without an offer of payment. They will be aware that preceding participants had received payments for completion of measures.

The end of the study will be defined as the date at which the participant debrief is distributed to all participants. This will take place after eligible participants have been invited to complete the second set of follow-up measures (April 2023).

## Statistical analytical plan

We will report data in line with the CONSORT 2010 Statement showing attrition rates and loss to follow-up (Schulz, et al., 2010). All analyses will be carried out using the intention to treat principle, incorporating data from all participants including those who do not complete the intervention. Every effort will be made to follow up all participants in both arms for outcome assessments. A detailed plan will be signed off prior to analysis, which will be performed in Stata 15 or higher.

### Primary and secondary outcome analysis

**Objective 1a**: Descriptive statistics within each randomised arm will be presented. At baseline these will include counts/percentages for binary and categorical variables, and means/standard deviations, or medians with lower and upper quartiles, for continuous variables, along with minimum and maximum values and counts of missing values. Outcomes at 6 months will be presented separately for each arm and summarised using counts/percentages for binary/categorical variables and means/standard deviations for continuous variables, along with counts of missing values. Number of adverse events will be presented as the number of events and number of individuals with events and will be provided separately for each randomised arm and according to the treatment received. We will also report the number of children and parents, in each arm, whose anxiety deteriorates.

To test primary and secondary outcomes, we will analyse using multiple linear regression and include a fixed effect for intervention vs waitlist, adjust for baseline child anxiety severity. Other covariates considered, a priori, to be prognostic of outcome at 6 months (particularly parent gender), may be included in the linear models and written into analysis plan prior to sign off. Treatment effects (between-group differences) will be reported as adjusted mean difference with 95% confidence intervals. Cohen’s D effect sizes at 6 months will be calculated as adjusted mean difference of outcome divided by sample standard deviation of the outcome at baseline. Potential moderators will be assessed by including randomised arm by moderator interactions as fixed effects.

**Objective 1b**: To test the active ingredients of the intervention, we will conduct a component analysis. There will be three randomised groups: those randomised to intervention containing a given Module (e.g., Module E), those randomised to intervention but not containing that component (e.g., Module E), and those randomised to the control arm (i.e., no intervention). Using the measure of parent behaviour congruent with each module as dependent variable, we will test whether there are differences between these groups for each of modules A to H separately. For those modules that demonstrate an effect, we will perform a mediation analysis with the primary outcome as dependent variable and the measure of parenting behaviour congruent with that module as a mediator.

**Objective 2:** A linear regression (ANCOVA) model will be fitted with 6-month data as the outcome, baseline measure of outcome as a covariate, and the randomisation variable as a fixed effect. We will fit a mixed effects model where additional follow-up data are also available and include a random effect for participant.

**Objective 3**: This analysis will be restricted to participants where the co-parent agrees to provide data. We will include baseline covariates (their anxiety type and severity; parenting variables) collected on them in a linear regression model with randomisation as a fixed effect and child anxiety severity at 6-months as the outcome variable.

### Interim analysis

Monthly checks on attrition levels across the intervention modules will be carried out. If a disproportionate level of attrition is associated with a particular module(s), this may be adapted, subject to discussion with the Trial Steering Committee (see 5.1 for additional information)

## Procedure to account for missing or spurious data

Given the expected high levels of attrition, we need to take great care to determine whether those who drop out are representative of those who remain in the study.

As per the CONSORT 2010 statement we will keep track of all participants, including those who stop the intervention early but remain happy to continue to provide follow-up data, and those who no longer wish to participate in the intervention or provide further follow-up data.  As a minimum, we will state the proportion retained at each time point, by trial group, and compare the characteristics of completers vs. non-completers. For the purpose of this study, a study completer will be defined as a participant who has accessed at least three modules.

For scale data with actively unanswered items, we will either pro-rate where sufficiently many (e.g. >80%) items are completed, or we will perform multiple imputation at the item-level.  We will perform multiple imputation if we can identify predictors of missingness upon fitting logistic regression models which include variables not in the analysis models, which may be variables collected post-randomisation.

Where outcome data are missing (assumed to be missing at random) we will perform multiple imputation using chained equations to create (e.g. 10) completed datasets, the analyses of which will be pooled using Rubin’s Rules.

# Data Management, Protection & Confidentiality

Data will be entered directly by participants via the online web platform Moodle and via an embedded Qualtrics survey.  Measures data is stored in pseudonymous form on Qualtrics servers, whilst other data-tables (scores) and the identity table (identifying information) exist together in a database on the AWS server.

The participants will be entering identifiable data when registering for participation in the study.  At randomisation, the participants will each be given a unique identifier by which they will be referred for the duration of the study. This *entire* database is encrypted. The application has been created with its own private server. In order to collect special category data (e.g. mental health data, ethnicity) we have put in place an SSL, which will establish an encrypted link between a web server and a browser. This link ensures that all data passed between the browser and web server remain private and integral. SSL is industry standard and used by millions of websites to protect online transactions with their customers. The database will be held by the CI, and programmer/database manager, who will control access to the data.

The same industry standard encryption practices (e.g. SSL and db encryption) apply to the Qualtrics database storing the measures.  Email addresses and names are not stored by the Qualtrics survey.

Data will not be shared outside the research group (although the anonymised dataset will be entered into a repository at a later date). We will store data downloaded from the website on password protected spreadsheets hosted on a University of Brighton or University of Sussex server. We will use a platform from a third party to deliver the digital intervention. Appropriate arrangements will be put in place to ensure adherence to data protection requirements. This platform is highly secure and designed for use in highly regulated environments including University and National Health Service settings and has accordingly high standards of encryption and data protection. The database will be checked regularly by the Trial Manager to ensure that complete sets of data are being collected and stored appropriately.

Data generated from the study will be analysed by members of the research team. When the data is extracted from the web platform for analysis, no identifiable data will be transferred. The extracted anonymised data will be analysed on password protected computers. The link to connect the identifiable participant information, and the anonymised data will be held by the Chief Investigator on password protected University of Sussex computers. The research data generated from the study will be stored for at least 5 years from the end of the study and then will be transferred for long-term storage within the University of Sussex data archive, Figshare.

Consent will be sought from each participant to allow anonymised sharing of research data for future research in this area.

# Ethical and regulatory considerations

Data collection will commence only after ethical and governance approval are obtained from the Sponsor. Informed consent will be obtained after participants have read and reviewed an approved information sheet. Since all participants will have self-referred, capacity to consent will be assumed.

# Participant Safety

International Conference for Harmonisation/Good Clinical Practice (ICH/GCP) requires Investigators and Sponsors to follow specific procedures when notifying and reporting adverse events/reactions in research studies.

In accordance with the University of Sussex Safeguarding policy (available at: <https://www.sussex.ac.uk/safeguarding/>) any concerns regarding children or adults who may be at risk should be reported promptly through the University *safeguarding* protocol and then if necessary, externally to the Local Authority.

For this study, there are no anticipated significant safety concerns: no adverse reactions were reported in a previous study of the face-to-face intervention. To capture any serious adverse events, and to protect affected participants, the study team will take a 4-pronged approach.

1. Signposting to external sources of assistance will be displayed prominently throughout participants’ journey through the study. Participants in the control group will be provided with the same resources when informed they are allocated to the control arm.
2. Participants will have an option to ‘contact the team’ This will enable an email to the trial manager. This email account will not be staffed at all times, but an auto-reply will direct participants who need it to immediate sources of help.
3. There is no standard method for recording of adverse events in psychoeducational studies, and methods for drug studies are inappropriate, increasing participant burden and distress and, therefore, attrition. An idiosyncratic approach, capturing the harms that are most likely in each individual study is probably the most appropriate approach (Duggan, Parry, McMurran, Davidson, & Dennis, 2014). In this study, these are: stress, anxiety, sadness, unpleasant memories, negative parental self-criticism. However, these are all fluctuating natural features of anxiety, so there is little value (and considerable cost) in measuring them frequently. Instead a brief questionnaire will assess these once, for both conditions, at the six-month outcome assessment point.
4. In a review of adverse-events capture in psychosocial studies, Duggan, Parry, McMurran, Davidson, & Dennis (2014), caution against the over-assessment of these. In line with their recommendations, we will report the number of parents and children, in each arm, whose own anxiety deteriorates. This will indicate the number of participants who experience potential harm as a result of the intervention.

If any adverse events deemed serious are identified by any member of the research team throughout the duration of the study, they will be reported to the Chief Investigator of the study immediately, the Sponsor, and the Clinical Trials Unit will also be notified at [BSCTUsafety@bsms.ac.uk](mailto:BSCTUsafety@bsms.ac.uk).

The CI will assess whether the event is:

a/ Serious (categorised by; resulting in death, life threatening, requiring inpatient hospitalisation or prolongation of existing hospitalisation, resulting in persistent or significant disability, resulting in congenital anomaly, other medically important event)

b/ Related to (i.e. caused by) the study intervention

c/ Expected due to the nature of the intervention

Relatedness criteria for this study: distress caused by the content of the intervention, distress caused by taking part in the study, disappointment at being allocated to the control arm, other as deemed by investigator.

## Research ethics committee and reports

The study will be approved by the University of Sussex Cross Schools Research Ethics Committee.

## Peer review

The study protocol was subject to peer review as a component of the original grant application process. In response to reviewer feedback the following amendments were made to the original protocol:

- The planned waitlist control design was changed to a no treatment control condition to enable the second round of follow-up assessments to have comparison with an untreated control group and to enable longer-term controlled follow-up studies.
- The invitation to refer a co-respondent was extended to all study participants and these assessments were extended to all follow-up data collections points. This was incorporated into a SWAT design.

## Public and Patient involvement

This project arose from focus groups with users of our service and included clinicians and parents. The two service-user representatives on this application have lived experience of parenting in the context of anxiety disorder and have both completed the face-to-face version of the digital intervention under scrutiny here. They will commit 1 day per month each for the duration of project. They will have strategic input, as full members of the Trial Management Group, but will also contribute to the day-to-day running of the project according to their skills and interests. For example, CEP has a degree in English and has expressed particular interest in our communication and dissemination work; NM has a business interest in social media and will take a key role in our recruitment process. Both will have oversight of all public-facing study materials. Budget is allocated for specialized training and conferences for our User Representatives.

## Post trial care

Participants will receive a debrief sheet once study outcome data collection is closed. This will thank participants for their involvement in the study, outline the objectives of the study, provide relevant preliminary results (e.g. number of participants), will inform participants of planned further research and provide information about appropriate sources of support.

# Dissemination policy

A protocol paper, and subsequent outcomes (reported in full) will be delivered to academic/clinical audiences via fully open-access journals, and conferences (four, including user-conferences). All team members (including user representatives) will be authors on all papers. SCH or the Trial Manager will lead on primary publications. KL will lead on the ‘prospective’ paper. Anonymised data will be entered into a data repository at end of study (in liaison with steering and ethics committees). Results will be communicated to participants and wider public via a short video. Working with the University media office and our PR company, we will issue press-releases. Plain English results will be disseminated to all stakeholders and participants, and appear on the study website.

Our close integration with Sussex Partnership Foundation Mental Health Trust and links with the local Clinical Commissioning Group will facilitate rapid dissemination to service providers. Our links with national charities and NICE will facilitate links to policy makers.

# Expected Outcomes of the Study

At the end of this study, we will have an evidence-based intervention for the prevention of anxiety in the children of a broad spectrum of anxious parents. We will have information that will enable us to optimise the intervention for maximum efficiency, and to begin tailoring it to the needs of individuals. As well as potentially reducing individual suffering in children, we anticipate that the intervention will lead to improvements in parental mental health.

The intervention will be suitable for rapid rollout and for very wide scale use, with minimal resource implications for providers or users.

Thanks to our unique, large longitudinal sample, we will have a better understanding of the role of the parent and co-parent in the intergenerational transmission of anxiety. This will have impacts not only on future preventive efforts, but also on family-based treatments for existing child anxiety disorders.

Finally, we will have added to the important evidence base on maximizing response rates in online trials, which could have widespread benefits across future RCTs in a range of domains.

# Description of the research group(s)/research environment(s)

**SCH** is a clinical psychologist and academic who developed the intervention under scrutiny here. She is Director of the Flourishing Families Clinic (a unique, NHS-based research clinic, focused on the prevention of mental health problems in children of adults attending adult mental health services). She has completed two RCTs within budget. She was awarded the British Psychological Society ‘May Davidson Award’ for her research in the first ten years of her career. She was part of the NICE Guideline panel for Social Anxiety Disorder.

**JS** is an experimental neuropsychologist of 20 years experience. She publishes in high impact journals including Nature, Trends in Cognitive Sciences, Proceedings of the Royal Society of London. She has held multi-million pound funding and has experience of conducting very large-scale online studies (100,000+). She designed the online web platform [syntoolkit.org](http://syntoolkit.org/) which will be used here, and which offers an innovative way to conduct online testing with the safety and rigour of local testing.

**KL** is an expert in the field of child anxiety and parenting. She has published widely on the cognitive and behavioural processes in intergenerational transmission of anxiety. She brings significant experience in intervention-based research for anxiety disorders, having coordinated a large international study of genetic and clinical predictors of response to CBT. Her recent research investigates the impact of mothers and fathers on children’s play experiences, with a particular focus on adventurous play and its effects on children’s social, emotional and cognitive development.

**RE** is an NIHR Research Professor and Professor of Medical Statistics and Trials Methodology at IOPPN and will act as collaborator. His research develops statistical methods for causal inference, and efficacy and mechanisms evaluation. Current applications include RCTs of complex interventions in mental health, and trial designs and associated analysis methods in precision medicine. He is lead for the Statistical Analysis Working Group in the NIHR-MRC Trials Methodology Research Partnership, and is currently trial statistician on 15 NIHR and MRC funded randomised trials.

**SB** has been a medical statistician for 23 years and working in RCTs since 2008. He has worked on numerous studies in mental health (trials and epidemiological studies). He is senior statistician co-applicant on three active research studies in mental health. He is also the independent statistician on one active and three completed trial oversight committees for trials in mental health.

**PL** will bring expertise in anxiety prevention (he led the meta-analytic study cited by the Kavli Trust in support of the anxiety prevention evidence gap); He is a clinical psychologist with experience of working on RCTs and has conducted prize-winning research into the risk factors for and prevention of anxiety disorders (British Psychological Society PsyPAG Rising Researcher awards, 2nd place; University of Reading, Division PhD Researcher of the Year).

**AD** is University of Sussex Research Fellow attached to the study. She is a research psychologist focused on the intersection of parental mental health and parenting. She has delivered the face-to-face version of the intervention. She will take part responsibility of day-to-day running of the trial with AA.

**AA** is a Trial Manager for the Brighton & Sussex Clinical Trials Unit. She has worked within a wide variety of clinical specialties, with over ten years’ experience in data management, trial monitoring and trial management.

**CEP** and **NM** are experts by experience, being both parents and having personal experience of anxiety disorders. Both have participated in the face-to-face version of this intervention.

The study will be formally supported by the **Brighton and Sussex Clinical Trials Unit**.

# Leadership

The project and its financial management will be overseen by the PI, who is experienced in delivering similar trials. A Trial Management Group, comprising the applicants plus the trial manager, will meet twice a year. A Steering Committee, appointed to NIHR guidelines, and including an independent chair, statistician and lay involvement, will also meet twice yearly. The team will have access to the research and professional development facilities available to employees of Brighton & Sussex Medical School and the Universities of Sussex and Southampton. Each institution has professional support mechanisms for research funding, IT support, ethics, public relations and governance.

# Funding and support

The trial is funded by the KAVLI Trust. Funding includes provision for a full-time postdoctoral trial manager, support from the BSUH Clinical Trials Unit, a technician to set up and manage the web platforms and for a public relations agency to support recruitment.

# References

Bailey, J. V, Pavlou, M., Copas, A., McCarthy, O., Carswell, K., Rait, G., … Murray, E. (2013). The Sexunzipped trial: optimizing the design of online randomized controlled trials. *Journal of Medical Internet Research*, *15*(12), e278. <https://doi.org/10.2196/jmir.2668>

Barker, B., Iles, J. E., & Ramchandani, P. G. (2017). Fathers, fathering and child psychopathology. *Current Opinion in Psychology*, *15*, 87–92. <https://doi.org/10.1016/J.COPSYC.2017.02.015>

Björgvinsson, T., Kertz, S. J., Bigda-Peyton, J. S., McCoy, K. L., & Aderka, I. M. (2013). Psychometric Properties of the CES-D-10 in a Psychiatric Sample. *Assessment*, *20*(4), 429–436.

Bögels, S. M., & Perotti, E. C. (2011). Does Father Know Best? A Formal Model of the Paternal Influence on Childhood Social Anxiety. *Journal of Child and Family Studies*, *20*(2), 171–181.

Bögels, S., & Phares, V. (2008). Fathers’ role in the etiology, prevention and treatment of child anxiety: A review and new model. *Clinical Psychology Review*, *28*(4), 539–558.

Brueton, V. C., Tierney, J. F., Stenning, S., Meredith, S., Harding, S., Nazareth, I., et al. (2014). Strategies to improve retention in randomised trials: a Cochrane systematic review and meta-analysis. *BMJ Open*, *4*(2), e003821.

Cartwright-Hatton, S., Ewing, D., Dash, S., Hughes, Z., Thompson, E. J., Hazell, C. M., … Startup, H. (2018). Preventing family transmission of anxiety: Feasibility RCT of a brief intervention for parents. *British Journal of Clinical Psychology*. *57*(3), 351-366

Cartwright-Hatton, S, Abeles, P., Dixon, C., Holliday, C., & Hills, B. (2013). Does parental anxiety cause biases in the processing of child-relevant threat material? *Psychology and Psychotherapy: Theory, Research and Practice*.

Cartwright-Hatton, Sam, McNally, D., Field, A. P., Rust, S., Laskey, B., Dixon, C., … Woodham, A. (2011). A New Parenting-Based Group Intervention for Young Anxious Children: Results of a Randomized Controlled Trial. *JAACAP*, *50*(3), 242-251.e6.

Cartwright-Hatton, S, McNicol, K., Doubleday, E. (2006). Anxiety in a neglected population:prevalence of anxiety disorders in pre-adolescent children. *Clinical Psychology Review*, *26*(7), 817–833.

Arundel C, Coleman E, Fairhurst C et al. SCIMITAR+ Trial: A randomised study within a trial (SWAT) of a contingent financial reward to improve trial follow-up [version 1; peer review: 1 approved, 1 not approved]. *F1000Research* 2019, **8**:1937 (<https://doi.org/10.12688/f1000research.21059.1>)

Duggan, C., Parry, G., McMurran, M., Davidson, K., & Dennis, J. (2014). The recording of adverse events from psychological treatments in clinical trials. *Trials*, *15*(1), 335.

Edwards, P. J., Clarke, M. J., Roberts, I., DiGuiseppi, C., Wentz, R., Kwan, I., … Pratap, S. (2009). Methods to increase response to postal and electronic questionnaires (Review). *The Cochrane Library*, *3*(3). <https://doi.org/10.1002/14651858.MR000008.pub4.www.cochranelibrary.com>

Eley, T. C., McAdams, T. A., Rijsdijk, F. V., Lichtenstein, P., Narusyte, J., Reiss, D., … Neiderhiser, J. M. (2015). The Intergenerational Transmission of Anxiety: A Children-of-Twins Study. *American Journal of Psychiatry*, appi.ajp.2015.1.

Field, A., Lester, K., Cartwright-Hatton, S., Harold, G., Shaw, D., Natsuaki, M., … Leve, L. (Under Review). Maternal and paternal influences on the developmental trajectory of childhood anxiety symptoms: a genetically sensitive investigation.

Galbraith, S., Bowden, J., & Mander, A. (2017). Accelerated longitudinal designs: Overview of modelling, power, costs & handling missing data. *Statistical Methods in Med Res,* *26*(1), 374-398.

Herdman M, Gudex C, Lloyd A, Janssen MF, Kind P, Parkin D, Bonsel G, Badia X (2011). Development and preliminary testing of the new five-level version of EQ-5D (EQ-5D-5L). Quality of Life Research 20(10):1727-36.

Lawrence, P. J., Murayama, K., & Creswell, C. (2019). Systematic Review and Meta-Analysis: Anxiety and Depressive Disorders in Offspring of Parents With Anxiety Disorders. *Journal of the American Academy of Child & Adolescent Psychiatry*, *58*(1), 46–60.

Lester, K. J., Field, A., & Cartwright-Hatton, S. (2012). Maternal anxiety and cognitive biases towards threat in their own and their child’s environment. *Journal of Family Psychology*, *26*(5), 756.

Lyneham, H. J., Sburlati, E. S., Abbott, M. J., Rapee, R. M., Hudson, J. L., Tolin, D. F., & Carlson, S. E. (2013). Psychometric properties of the Child Anxiety Life Interference Scale (CALIS). *Journal of Anxiety Disorders*, *27*(7), 711–719. <https://doi.org/10.1016/j.janxdis.2013.09.008>

Majdandžić, M., de Vente, W., & Bögels, S. M. (2016). Challenging Parenting Behavior from Infancy to Toddlerhood: Differences between Fathers and Mothers. *Infancy*, *21*(4), 423–452.

Majdandžić, M., Lazarus, R. S., Oort, F. J., van der Sluis, C., Dodd, H. F., Morris, T. M., … Bögels, S. M. (2018). The Structure of Challenging Parenting Behavior and Associations With Anxiety in Dutch and Australian Children. *Journal of Clinical Child & Adolescent Psychology*, *47*(2), 282–295.

Murphy, J. M., Bergmann, P., Chiang, C., Sturner, R., Howard, B., Abel, M. R., & Jellinek, M. (2016). The PSC-17: Subscale scores, reliability, and factor structure in a new national sample. *Pediatrics*, *138*(3). https://doi.org/10.1542/peds.2016-0038

Panter-Brick, C., Burgess, A., Eggerman, M., Mcallister, F., Pruett, K., & Leckman, J. F. (2014). Practitioner Review: Engaging fathers-recommendations for a game change in parenting interventions based on a systematic review of the global evidence.

Ramchandani, P., & Psychogiou, L. (2009). Paternal psychiatric disorders and children’s psychosocial development. *The Lancet*, *374*(9690), 646–653.

Lawrence, P., Rooke, S.M. & Creswell, C. (2017). Prevention of anxiety among at-risk children and adolescents a systematic review and meta-analysis. *Child & Adolescent Mental Health,* *22*, 118-130.

Schulz, K. F., Altman, D. G., & Moher, D. (2010). CONSORT 2010 Statement: Updated guidelines for reporting parallel group randomised trials. *BMJ (Online)*, *340*(7748), 698–702. <https://doi.org/10.1136/bmj.c332>

Spence, S H. (1997). Structure of anxiety symptoms among children: A confirmatory factor-analytic study. *Journal of Abnormal Psychology*, *106*(2), 280-297.

Spence, Susan H, Rapee, R., McDonald, C., & Ingram, M. (2001). The structure of anxiety symptoms among preschoolers. *Behaviour Research and Therapy*, *39*(11), 1293–1316.

Spitzer, R. L., Kroenke, K., Williams, J. B. W., & Löwe, B. (2006). A Brief Measure for Assessing Generalized Anxiety Disorder. *Archives of Internal Medicine*, *166*(10), 1092.

Stewart-Brown, S., Tennant, A., Tennant, R., Platt, S., Parkinson, J., & Weich, S. (2009). Internal construct validity of the Warwick-Edinburgh Mental Well-Being Scale (WEMWBS): A Rasch analysis using data from the Scottish Health Education Population Survey. *Health and Quality of Life Outcomes*, *7*(1), 15. <https://doi.org/10.1186/1477-7525-7-15>

van Steensel, F. J. A., & Bögels, S. M. (2014). An adult version of the Screen for Child Anxiety Related Emotional Disorders (SCARED-A). *Netherlands Journal of Psychology*, *68*.

Wille, N., Badia, X., Bonsel, G., Burström, K., Cavrini, G., Devlin, N., Egmar, A. C., Greiner, W., Gusi, N., Herdman, M., Jelsma, J., Kind, P., Scalone, L., & Ravens-Sieberer, U. (2010). Development of the EQ-5D-Y: a child-friendly version of the EQ-5D. *Quality of life research : an international journal of quality of life aspects of treatment, care and rehabilitation*, *19*(6), 875–886. <https://doi.org/10.1007/s11136-010-9648-y>

# Appendices

**Digital Intervention Content**

The intervention comprises the following elements:

**Starter Module**. *All about anxiety & confidence in children: Cognition; Fight flight; Avoidance.*

In this module, parents are given a basic understanding of the psychological processes that underpin anxiety. Upon completion of this module, they will understand the basic physiological and cognitive underpinnings of the fight-flight process. They will be aware that the physical symptoms of anxiety are (in moderate amounts, at least) not harmful to their child. They will understand the critical role of avoidance in the development and maintenance of anxiety and will have some basic strategies for reducing avoidance behaviour and increasing approach behaviour in their children. They will have a basic understanding of the type of thoughts that are problematic for anxious children and of the confident thoughts that would be expected in a healthy child. These ‘Seven Confident Thoughts’ form the framework upon which the rest of the intervention is hung.

**Module A:** *The role of avoidance and small steps to reducing it.* This module teaches parents about the role of avoidance in the development and maintenance of anxious symptoms. Parents are taught how to break a large fear into small, manageable steps that their child can approach little by little. By the end of the module, parents will understand that the easiest way to tackle a large fear (in children or in themselves) is to break it into smaller steps – i.e. a ‘fear hierarchy’. They will know how to draw up a ‘fear hierarchy’, and if appropriate (i.e. if their child has a current phobia) they will have drawn one up for their child. They will also know how to use this fear hierarchy to help a child to challenge their phobia (i.e. pacing of exposure, what to say during exposure (and what not to say), what to do if exposure does not go to plan, how to motivate the child).

**Module B.** *Using play to develop children’s confidence*. In this module, parents are taught about the importance of play (of various different kinds) for children’s mental health. By the end of the module, parents will understand the importance of: play with parents, play with siblings, play with peers, adventurous play, outdoor play, rough and tumble play. They will have assessed their children’s current access to each type of play, and will have a written plan for increasing their children’s opportunity for these types of play, if any are currently limited.

**Module C.** *Using ‘Emotion Coaching’ with children*. In this module, parents learn to tune into their children’s varied emotions, and to respond positively to each of these. By the end of the module, parents will understand the importance of noticing emotion in their children. They will know that is important to help children to label their emotions and will have the vocabulary to do this. They will be aware of the need to problem solve emotions, alongside their child, and will know how to do this. Critically, at the end of this session, they will be aware that all emotions are acceptable in their child and are quite safe.

**Module D.** *Managing difficult behaviour – praise and reward.* Almost all children display difficult or unwanted behaviour at times, and parental management of this is an important predictor of children’s mental health. This session teaches parents how to manage difficult behaviour in a fair, gentle, and consistent manner. By the end of the session parents will have learned how to use praise to increase prosocial and confident behaviour in their children. They will have learned how to use rewards (in small doses) to help manage particularly tricky behaviours.

**Module E.** *The role of sleep, exercise and diet in children’s mental health*. In this module, parents learn the importance of sleep, exercise and diet for children’s mental health. By the end of the module, parents will have an indication of whether their child is getting enough sleep and will have a written plan for improving their children’s sleep quality. They will have an indication of whether their child is getting enough exercise for optimal mental health, and if not, will have a written plan for increasing this. The intervention does not go into great detail on children’s diet, but by the end of the module, parents will understand that if their child is to attempt something that is scary for them, then this will work better if they have recently eaten something.

**Module F.** Parenting hotspot: *Reducing Overprotection*. In this module, parents learn about the risks associated with overprotecting children. By the end of the module, they will have had the opportunity to consider whether they may be at risk of overprotecting their children. They will also have learned some techniques for becoming less overprotective, and have a written plan for applying these.

**Module G.** *Modelling confident behaviour; compensating for parenting gaps*. In this module, parents will find out how children can learn to be anxious or confident by watching their parents and other adults. By the end of the module, parents will be aware of the need to model confident behaviour wherever possible, and to minimise their children’s exposure to parental fears and worries. Parents will have been helped to identify any gaps in their children’s experience that have arisen as a result of parental anxiety (e.g. child has never been on a bus because of parental agoraphobia; child has rarely been to a large social gathering, because of parental social anxiety). They will have a written plan for filling these, gaps, using help from others where appropriate (e.g. family friend can take the child on buses; child can go to large family gathering with grandparents).

**Module H:** *Managing difficult behaviour: consequences and limit setting.* After completing this module parents have learnt how to set clear boundaries on children’s behaviour, and how to respond when children breach these boundaries, using logical and, where possible, natural consequences.

**Measures for Parenting with Anxiety Trial**

- Demographics Parent (Baseline)
  - Q5 repeated at 6m FU
- Demographics Child (Baseline)
  - Q5 repeated at 6m FU
- SPENCE (BL and FU)
- CSC-17 (BL and FU)
- EQ-5D-Y (BL and FU)
- CPBQ (BL and FU)
- ‘Your experience of being a parent’ Mediator measures. These are measures produced by trial team. (BL and FU)
- SCARED-A (BL and FU)
- SWEMWBS (BL and FU)
- GAD-7 (co-respondent measures) (BL and FU)
- Distress Questionnaire - parents (at 6 Month Follow-Up only)
- Distress Questionnaire – co-parents (at 6 Month Follow-Up only)
- Course Evaluation Questionnaire (for participants in the intervention arm at 6 Month Follow-Up only)

**Parent demographics**

What is your date of birth? (DD/MM/YYYY) [captured sign-in page]

1. At birth were you described as: Male □ Female □ Intersex □ I Prefer not to say □
2. Which of the following describes how you think of yourself? Male □ Female □ In another way □

2a) If you selected in another way, please describe……………………………………………………………

1. Which of these best describes your ethnic group?

White:

- English / Welsh / Scottish / Northern Irish / British
- Irish
- Gypsy or Irish Traveller
- Any other White background

Mixed / Multiple ethnic groups

- White and Black Caribbean
- White and Black African
- White and Asian
- Any other Mixed / Multiple ethnic background

Asian / Asian British

- Indian
- Pakistani
- Bangladeshi
- Chinese
- Any other Asian background

Black / African / Caribbean / Black British

- African
- Caribbean
- Any other Black / African / Caribbean background

Other ethnic group

- Arab
- Any other ethnic group

3a) If you ticked other, please describe: ……………………………………………………………………………..

1. How would you describe your financial status? Comfortable □ managing □ struggling □
2. Have you received treatment for anxiety in the last 12 months? Yes □ No □
3. Please indicate which of the following best describes when you left education

Left school before 16 □ Left school at 16 □

Left school 17/18 □ Completed college □

Completed university □

7) Information about your children

We will be asking you to answer some questions about one of your children. If you have more than one child aged between 2 and 11 the computer will choose one at random.

| Age | First name |
| --- | --- |
|  |  |
|  |  |
|  |  |
|  |  |
|  |  |

[Option to add more cells for larger family]

8) Do you live with the parent of [insert name of index child] Y/N

If no..

9) Would you classify yourself as a single parent household [are you the lone parent [birth/adoptive/foster/step] living in the house with your children]

**Child demographics**

For the following questionnaire and any other times we ask you to respond about your child please refer to [x]. If you have more than one child aged between 2 and 11 we have chosen one of these children at random to be the one you focus your answers on.

1. At birth was [insert name] described as: Male □   Female □   Intersex □   I Prefer not to say □

1. Which of these best describes your child’s ethnic group?

White:

- English / Welsh / Scottish / Northern Irish / British
- Irish
- Gypsy or Irish Traveller
- Any other White background

Mixed / Multiple ethnic groups

- White and Black Caribbean
- White and Black African
- White and Asian
- Any other Mixed / Multiple ethnic background

Asian / Asian British

- Indian
- Pakistani
- Bangladeshi
- Chinese
- Any other Asian background

Black / African / Caribbean / Black British

- African
- Caribbean
- Any other Black / African / Caribbean background

Other ethnic group

- Arab
- Any other ethnic group

3a) If you ticked other, please describe: ……………………………………………………………………………..

4.) Would you describe [insert name] as having a developmental disability?

This means your child has a severe and long-term condition which can relate to physical and/or mental functioning, including learning, speech and language or behaviour. Examples include Autistic Spectrum Disorder, Learning Disabilities and Down Syndrome.

5) Has [child] received treatment for anxiety in the last 12 months Yes □ No □

NB at 6month and subsequent follow-up this question will be repeated as below

"Has [child] had any other treatment for anxiety since you joined this study?" Yes □ No □

**Spence Children’s Anxiety Scale (SCAS)**


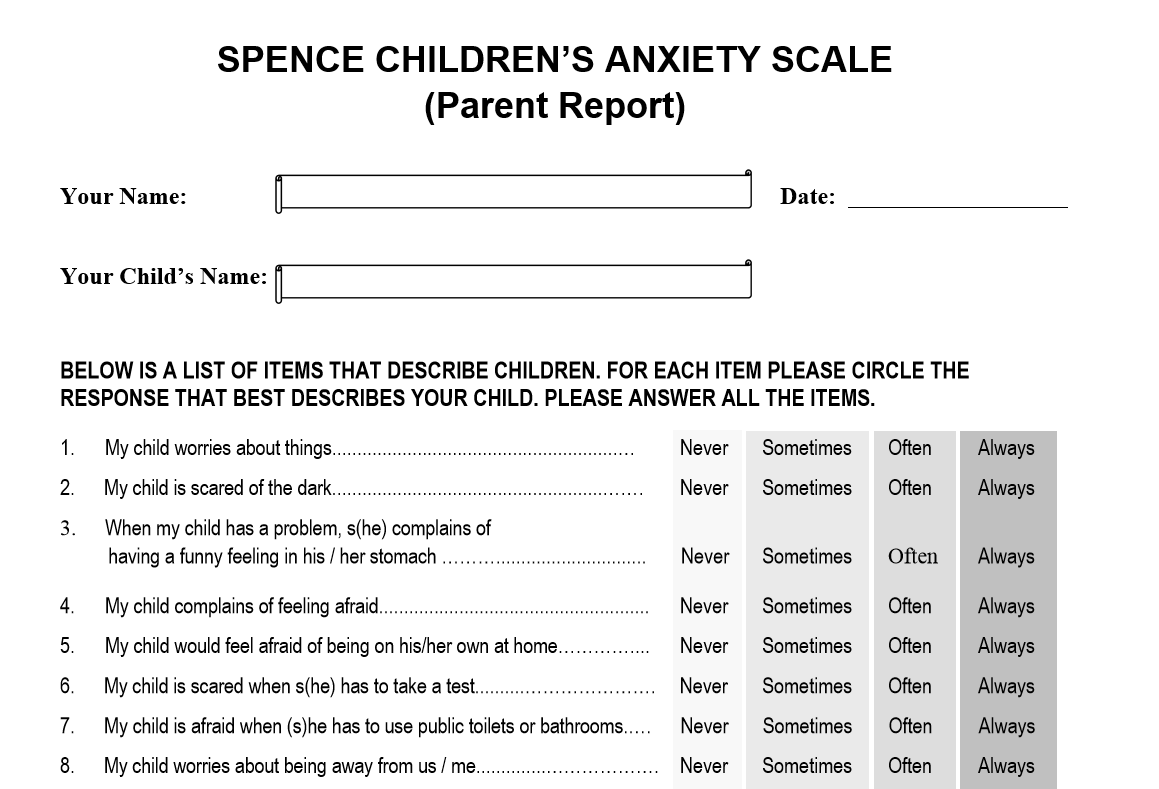

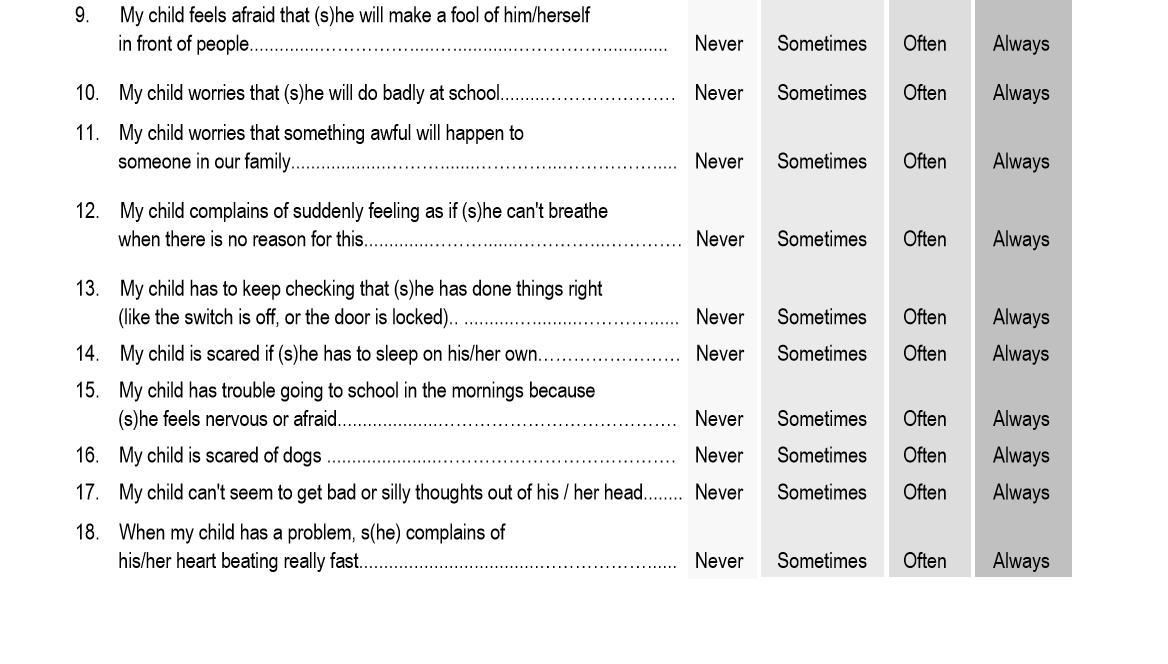


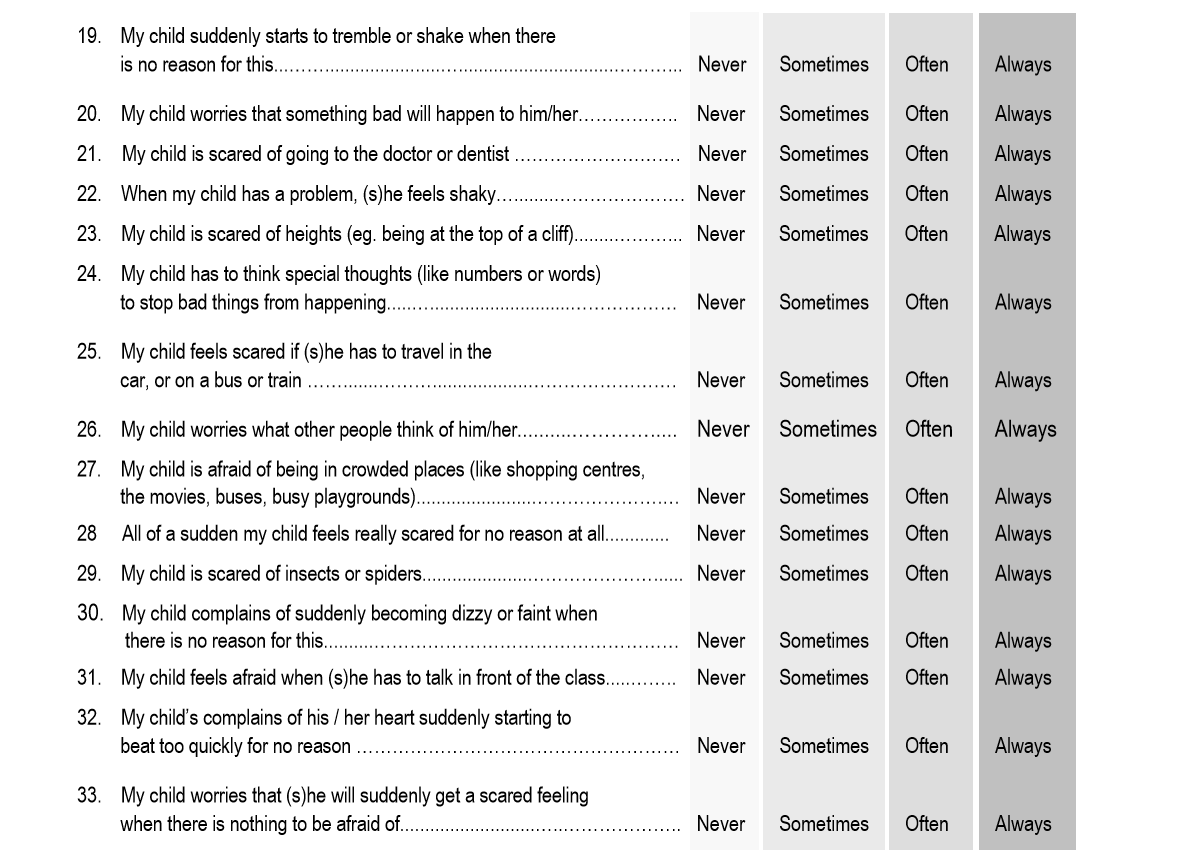


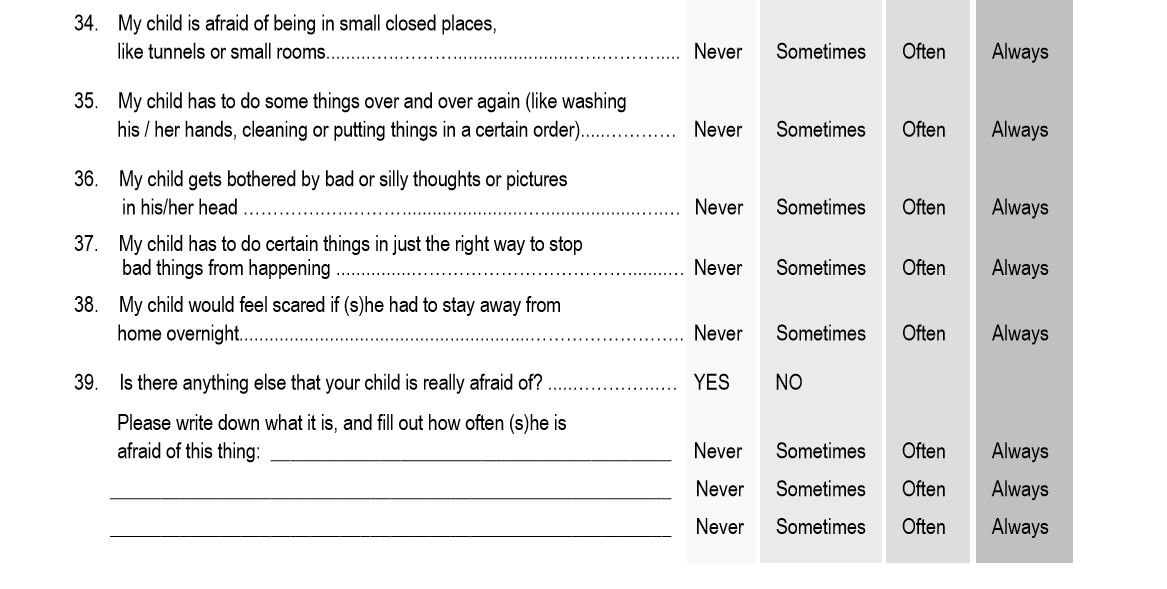


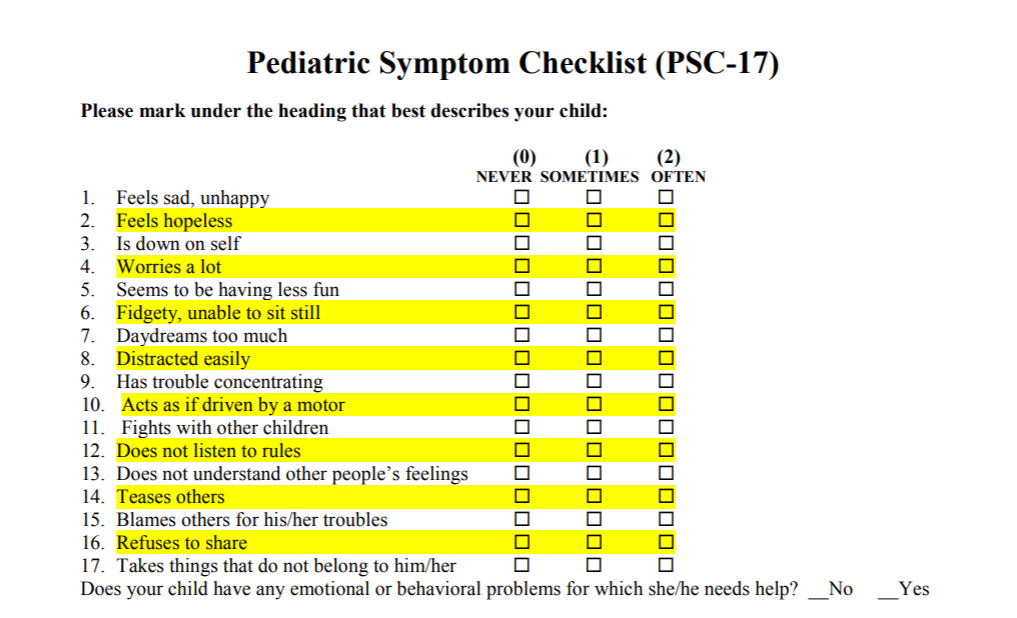


**EQ-5D-Y Proxy**

| Please select the ONE box that you think best describes the child's health TODAY. You should not answer on behalf of the child, but rather rate the child’s health as you see it. |
| --- |
| **MOBILITY** *(walking about)* |
| No problems walking about |
| Some problems walking about |
| A lot of problems walking about |
| **LOOKING AFTER HIM/HERSELF** |
| No problems washing or dressing him/herself |
| Some problems washing or dressing him/herself |
| A lot of problems washing or dressing him/herself |
| **DOING USUAL ACTIVITIES** (*for example: going to school, hobbies, sports, playing, doing things with family or friends)* |
| No problems doing his/her usual activities |
| Some problems doing his/her usual activities |
| A lot of problems doing his/her usual activities |
| **HAVING PAIN OR DISCOMFORT** |
| No pain or discomfort |
| Some pain or discomfort |
| A lot of pain or discomfort |
| **FEELING WORRIED, SAD OR UNHAPPY** |
| Not worried, sad or unhappy |
| A bit worried, sad or unhappy |
| Very worried, sad or unhappy |
| **We would like to know how good or bad you think the child’s health is TODAY.** |
| You will see a scale numbered from 0 to 100. |
| 100 means the best health you can imagine. 0 means the worst health you can imagine. |
| Please indicate on the scale how you think the child’s health is TODAY. |
| THE CHILD'S HEALTH TODAY = |
|  |
|  |

**Comprehensive Parenting Behavior Questionnaire 2-11 years**

*The statements below deal with how you interact with your child. Please circle a number to indicate to what degree the statements apply to you. If you are unsure, circle the number that represents the closest answer. Please ensure you answer all questions.*

| **1**  **Not**  applicable | **2**  **Slightly** applicable | **3**  **Sometimes, but sometimes not** applicable | **4**  **Usually** applicable | **5**  **Completely** applicable |
| --- | --- | --- | --- | --- |

1. I play little tricks on my child
2. I constantly keep an eye on my child, to prevent him/her from getting hurt
3. I regularly play or talk with my child for at least 5 minutes, with full concentration on each other, just for fun
4. I give my child the feeling that he/she is a burden on me
5. When my child really misbehaves, I spank him/her
6. When my child doesn’t listen to me, I explain that I find that annoying
7. I play boisterously with my child
8. I do not look at my child when he/she has disappointed me
9. I show my child that I love him/her
10. I am often harsh towards my child
11. My child knows how to persuade me not to give out punishment when he/she has done something wrong
12. I reward my child or give him/her something extra if he/she behaves well
13. If my child finds something scary, I encourage him/her to carry on regardless
14. I never take my child to busy places
15. I give my child the feeling that I love him/her the way he/she is; I don’t want to change him/her
16. At times I totally have had it with my child, and reject him/her
17. When my child whimpers or moans, I shout at him/her
18. When we go out, I prepare my child for this in advance
19. I encourage my child to approach unfamiliar people to ask them something
20. I cannot stand it when my child plays with things which make the house messy
21. I know exactly how to calm my child down when he/she is upset
22. I sometimes get very irritated when my child cries, and I do not hide it
23. I am often inconsistent in applying the rules I have made for my child
24. When my child has hit someone, I explain that he/she has hurt the other person
25. I encourage my child to be the best
26. I keep my child away from risky situations
27. I do fun activities with my child, such as handicraft or baking cookies
28. I am not easily pleased by my child
29. When I’m tense or irritable, I take it out on my child
30. I praise my child if he/she is being nice to other children
31. My child sometimes sees me horsing around with other people
32. I encourage my child to do things by him/herself
33. I show my love to my child by cuddling him/her, holding him/her and kissing him/her
34. When my child does something stupid, I react with irritation
35. When my child does not do what I ask, I often leave it at that
36. I make sure my child knows what is allowed and what is not
37. I regularly tease my child for fun
38. I don’t take my child out shopping because the fuss is too much for him/her
39. If my child does something naughty, I correct him/her, but at the same time I show that I still love him/her
40. When my child reacts differently from what I expected, I respond with disappointment
41. If my child does not do what I ask, even after repeated warnings, I slap him/her
42. When my child wants to touch something, he/she is not allowed to, I explain why it is not allowed
43. I almost never play rough and rowdy games with my child
44. If my child has hurt my feelings, I stop talking to him/her until he/she does me a favor
45. I comfort my child and show understanding when he/she is upset
46. My child hardly ever irritates me
47. I threaten to punish my child but then I fail to follow through
48. I reward or praise my child when he/she behaves properly, such as when he/she says ‘thank you’
49. If I see something that is new or exciting to my child, I encourage him/her to approach it
50. When my child climbs or clambers, I tell him/her that he/she should not climb too high because otherwise he/she might fall
51. Every day I play with child for a while, for example building something together or doing puzzles
52. When my child whimpers or moans, I shout at him/her
53. I set clear rules for my child
54. I encourage my child to perform for an audience by, for example singing a song, dancing, or doing something sporty
55. When I have a certain schedule in my head, my child has to cooperate
56. I often stroke my child’s head
57. When my child does something I don’t like, I often let it go
58. When my child has acted badly towards someone, I explain that it makes the other person sad
59. I challenge my child to contests, for instance running races or arm wrestling
60. I do not plan more than one outdoor activity per day because it might be too much for my child
61. If my son wants to dress up like a princess or my daughter wants to dress up like a pirate, I let him/her go ahead
62. When I’m stressed or tired, I react more severely to my child’s difficult behaviour
63. When my child helps, for example with clearing up toys, I give him/her a compliment
64. I show my child that I take risks
65. I encourage my child to do things in his/her own way
66. When my child asks something, I take my time when answering the question
67. The punishment I give my child depends on my mood
68. I give my child a warning when he\she appears to be starting to misbehave
69. As a prank, I sometimes give my child a real scare
70. During a risky activity I tell my child he/she should be careful
71. I often talk with my child
72. When my child goes too far, I slap him/her
73. I enjoy having pillow-fights with my child
74. When my child does something that’s not allowed, I pretend that he/she is not around
75. I often tell my child that I love him/her
76. When I ask my child to clean up and he/she does not do it, I eventually clean up myself
77. I encourage my child to do exciting things, such as jumping off high objects or climbing higher than he/she dares
78. I avoid doing things with my child that disturb his/her routine, for example when it means that bedtime will be later
79. I sometimes find it difficult to fully accept my child, including, his/her bad characteristics
80. If my child lingers when we are in a hurry, I shout at him/her
81. I encourage my child to say no if he/she doesn’t want something
82. I cannot stand it when my child suddenly wants something different from what we had planned
83. I immediately notice whether my child likes something or not
84. If my child makes a fuss when I say ‘no’, I give in to him/her
85. I encourage my child to compete against other children
86. I encourage my child to make his/her own decisions
87. When my child is naughty and I am stressed, I shout at him/her
88. My child often sees me approach unfamiliar people
89. When my child does something that is not allowed, I usually refuse to talk to him/her until he/she behaves better
90. When I ask my child to clean up and he/she does not do it, I eventually clean up myself
91. I pretend that I’m going to eat my child’s sweets, for example his/her cookies or dessert
92. I want to monitor everything that my child is doing
93. When my child misbehaves, I grasp him/her roughly
94. I sometimes play ‘tag’ with my child: I chase after him/her and say in a low voice that I’m going to grab him/her
95. I ask my child for his/her opinion about things
96. When I have said that my child is not allowed to do something, I stick to it
97. I encourage my child to gain new experiences by, for example, eating something new or playing a new game
98. I often try to get my child to change
99. When my child misbehaves in public, I scold him/her
100. I encourage my child to stand up for himself/herself
101. When my child does not listen, I respond without getting angry
102. I urge my child on when he/she is competing against other children
103. I show my child that I engage with situations that I find exciting or scary
104. If my child comes to me because he/she is having a minor quarrel, I make him/her sort it out by himself/herself

**Your experience of being a parent**

| Module | Item | Scale | Origin |
| --- | --- | --- | --- |
| Core |  |  |  |
| 1 | It is important that my child believes the world is a fairly safe place. | Strongly disagree, somewhat disagree, neither agree nor disagree, somewhat agree, strongly agree | Study |
| 2 | It is important that my child believes he/she can cope with most things | Strongly disagree, somewhat disagree, neither agree nor disagree, somewhat agree, strongly agree | Study |
| 3 | It is important that my child believes bad things don’t usually happen to them | Strongly disagree, somewhat disagree, neither agree nor disagree, somewhat agree, strongly agree | Study |
| 4 | It is important that my child believes bad things don’t usually pop up out of the blue | Strongly disagree, somewhat disagree, neither agree nor disagree, somewhat agree, strongly agree | Study |
| 5 | It is important that my child believes he/she has some control over the things that happen to them | Strongly disagree, somewhat disagree, neither agree nor disagree, somewhat agree, strongly agree | Study |
| 6 | It is important that my child believes people are pretty nice really | Strongly disagree, somewhat disagree, neither agree nor disagree, somewhat agree, strongly agree | Study |
| 7 | It is important that my child believes other people respect them | Strongly disagree, somewhat disagree, neither agree nor disagree, somewhat agree, strongly agree | Study |
| 8 | I believe that the world is a fairly safe place | Strongly disagree, somewhat disagree, neither agree nor disagree, somewhat agree, strongly agree | Study |
| 9 | It is OK for my child to be scared sometimes | Strongly disagree, somewhat disagree, neither agree nor disagree, somewhat agree, strongly agree | Study |
| Topic A  Avoidance |  |  |  |
| 1 | It is OK for my child to avoid things that frighten them | Strongly disagree, somewhat disagree, neither agree nor disagree, somewhat agree, strongly agree | Study |
| 2 | If my child became really scared of something I would know how to do a bravery ladder with them | Strongly disagree, somewhat disagree, neither agree nor disagree, somewhat agree, strongly agree, | Study |
| Topic B  Play |  |  |  |
| 1 | I prefer that my child is supervised closely by an adult when they are engaging in risky play | Strongly disagree, somewhat disagree, neither agree nor disagree, somewhat agree, strongly agree | Study: Amended from Toni |
| 2 | I stop my child from doing rough play | Strongly disagree, somewhat disagree, neither agree nor disagree, somewhat agree, strongly agree | Study |
| Topic C  Emotion Coaching |  |  |  |
| 1 | It is OK for my child to be angry or sad sometimes | Strongly disagree, somewhat disagree, neither agree nor disagree, somewhat agree, strongly agree | Study |
| 2 | I notice my child’s feelings and talk to them about it | Strongly disagree, somewhat disagree, neither agree nor disagree, somewhat agree, strongly agree | Study |
| 3 | I can cope when my child is upset | Strongly disagree, somewhat disagree, neither agree nor disagree, somewhat agree, strongly agree | Study |
| Topic D  +positive behaviour management |  |  |  |
| 1 | I reward or praise my child when he/she is brave, such a when he/she faces up to something they are scared of | Always/ very often/ sometimes/ rarely/ never | Study |
| 2 | I know how to use a star chart to help my child with a difficult behaviour | Strongly disagree, somewhat disagree, neither agree nor disagree, somewhat agree, strongly agree | Study |
| 3 | When I praise or reward my child, I tell them clearly what it is for. | Always/ very often/ sometimes/ rarely/ never | Study |
| 4 | Sometimes I will take away a reward if my child is naughty after I’ve given it. | Always/ very often/ sometimes/ rarely/ never | Study |
|  |  |  |  |
| Topic E  Basic needs/hacks |  |  |  |
| 1 | My child gets enough sleep | Every day, most days, occasionally, never | Study |
| 2 | My child gets enough exercise | Every day, most days, occasionally, never | Study |
| 3 | My child has caffeine (coffee, tea, cola, energy drinks) | Every day, most days, occasionally, never | Study |
| 4 | My child has a healthy diet | Every day, most days, occasionally, never | Study |
| Topic F  Hotspots & Overprotection |  |  |  |
| 1 | I try to be a perfect parent. | Strongly disagree, somewhat disagree, neither agree nor disagree, somewhat agree, strongly agree | Study |
| 2 | It is OK for my child to be a little stressed at times. | Strongly disagree, somewhat disagree, neither agree nor disagree, somewhat agree, strongly agree | Study |
| 3 | I let my child fail at things sometime | Strongly disagree, somewhat disagree, neither agree nor disagree, somewhat agree, strongly agree | Study |
| 4 | I am very protective of my child | Strongly disagree, somewhat disagree, neither agree nor disagree, somewhat agree, strongly agree | Study |
| Topic G  Modelling & Compensation |  |  |  |
| 1 | My child sees me feeling very frightened of things | Always/ very often/ sometimes/ rarely/ never | Study |
| 2 | I try to hide my anxiety from my child | Always/ very often/ sometimes/ rarely/ never | Study |
| 3 | If my child misses out on things because of my anxiety, I make sure they have those experiences in other ways | Strongly disagree, somewhat disagree, neither agree nor disagree, somewhat agree, strongly agree, not applicable | Study |


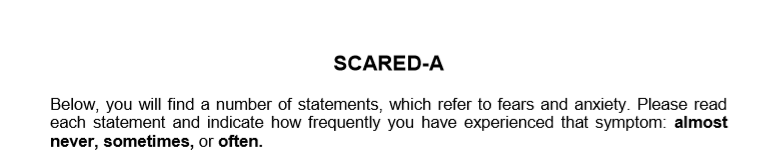

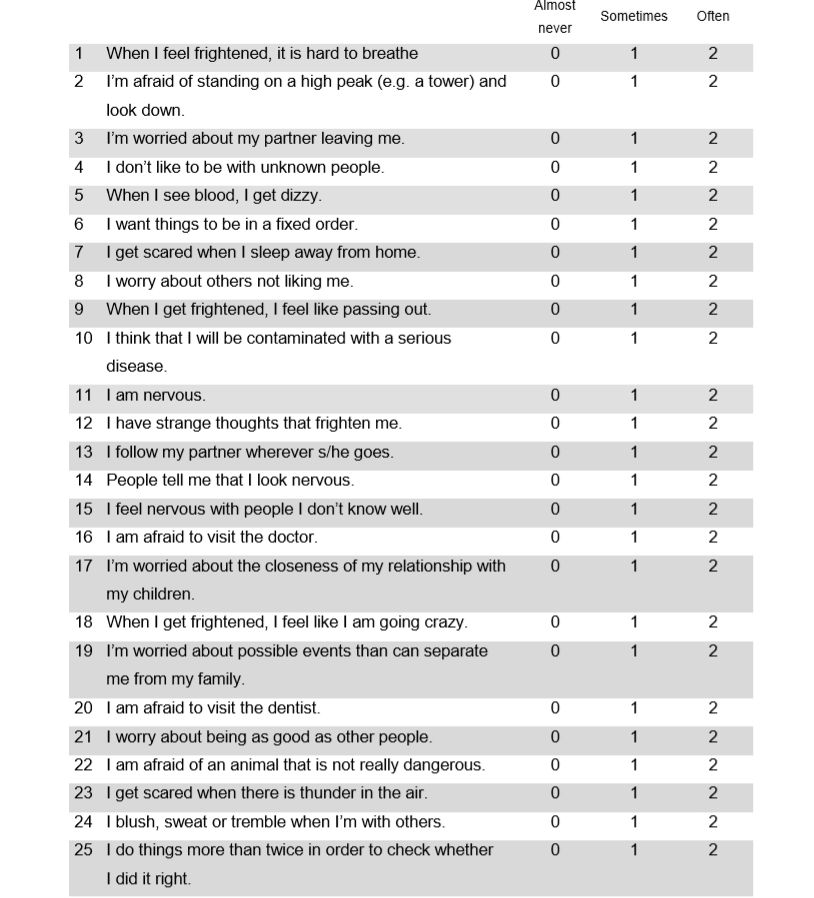


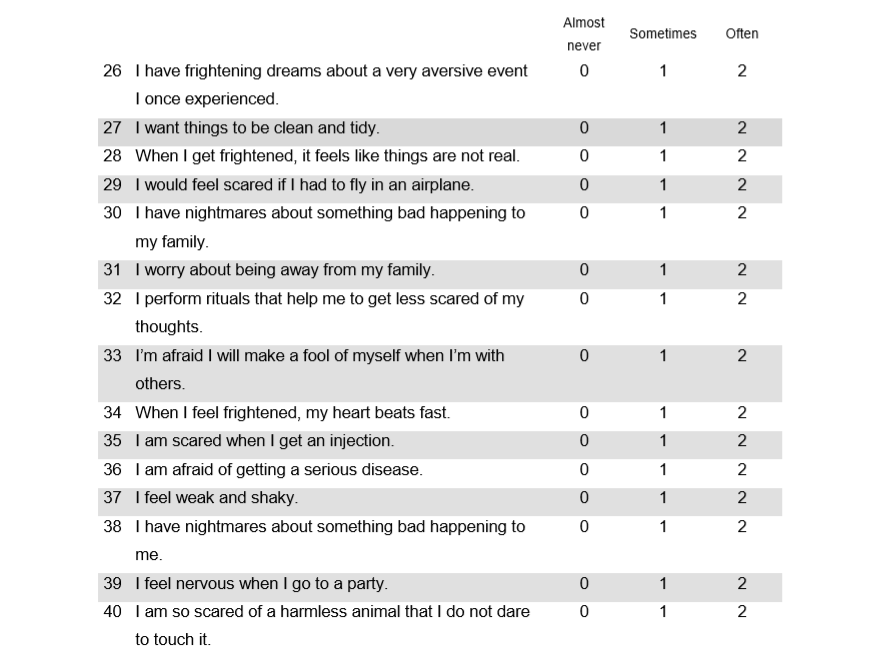

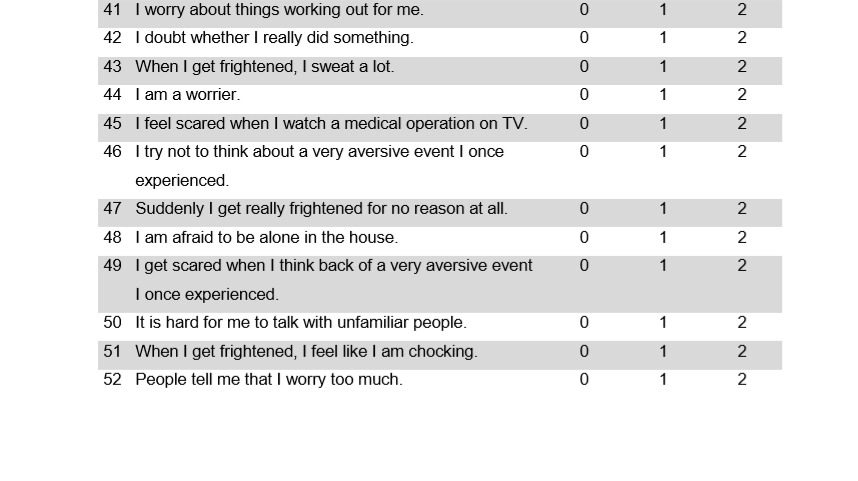

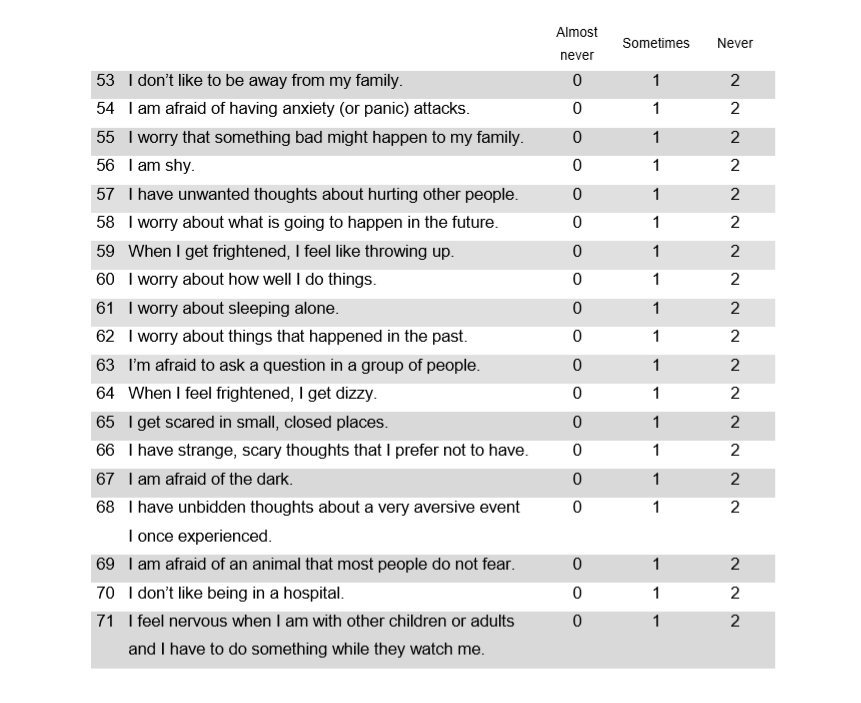


**SWEMWBS**


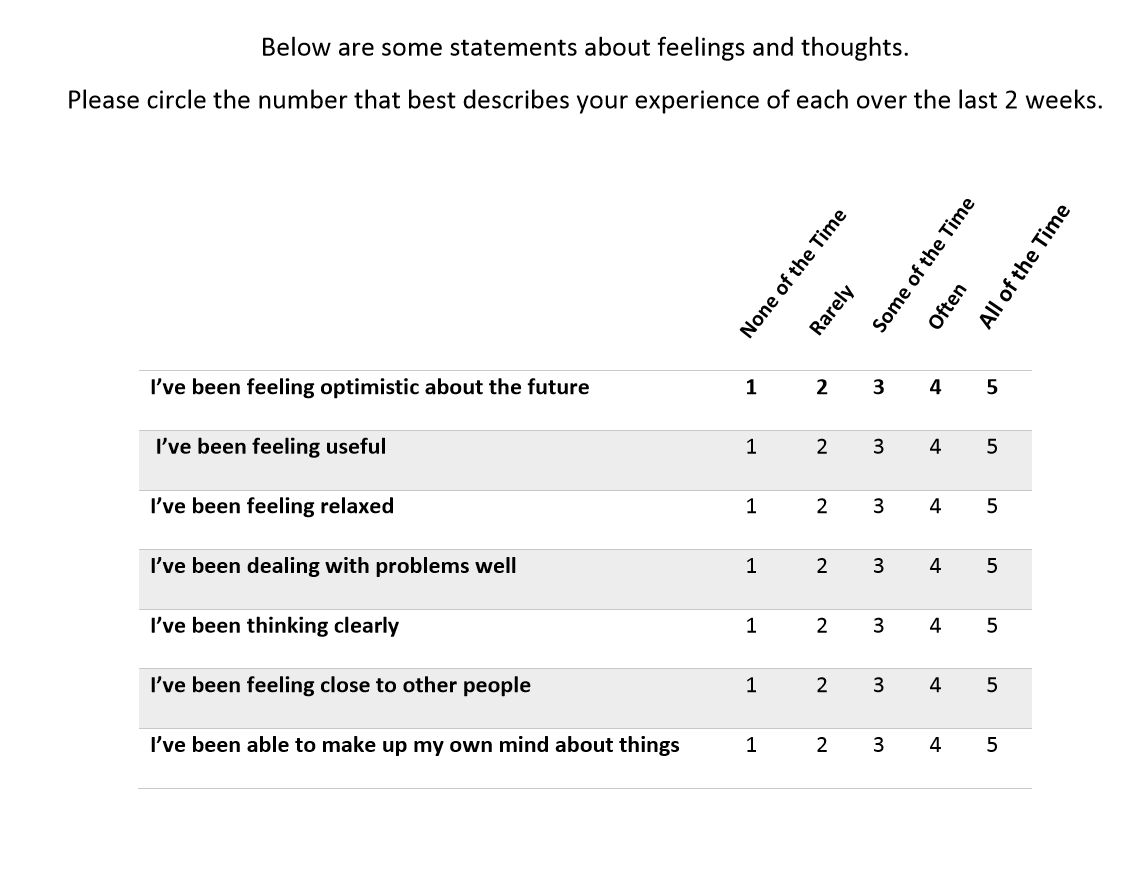


**Distress questionnaire for parents**

Administered in 6 month questionnaire pack (final question)

1. **Has taking part in the Parenting with Anxiety study made you feel upset or distressed**:

By the content of the course [Y/N/Don’t want to answer] (If Intervention arm only)

By taking part in the research study [Y/N/Don’t want to answer]

By being in the control group [Y/N/Don’t want to answer]  (If Control arm only)

1. **2. Has taking part in the Parenting with Anxiety study made you feel increased**:

Sadness [Y/N/Don’t want to answer]

Stress [Y/N/Don’t want to answer]

Anxiety [Y/N/Don’t want to answer]

Self-criticism [Y/N/Don’t want to answer]

Unpleasant memories [Y/N/Don’t want to answer]

**Distress questionnaire for co-parents**

Administered in 6 month questionnaire pack (final question)

1. **Has taking part in the Parenting with Anxiety study made you feel upset or distressed**:

[Y/N/Don’t want to answer]

1. **Has taking part in the PWA study made you feel increased:**

Sadness [Y/N/Don’t want to answer]

Stress [Y/N/Don’t want to answer]

Anxiety [Y/N/Don’t want to answer]

Self-criticism [Y/N/Don’t want to answer]

Unpleasant memories [Y/N/Don’t want to answer]

**Course evaluation questions (for participants on the intervention arm)**

1. Thinking about the course, please tell us which parts you found most useful.
2. Do you have any comments about the course you are willing to share with is?

GAD-7 Anxiety

| **Over the last 2 weeks, how often have you   been bothered by the following problems?**  *(Use “✔” to indicate your answer”* | Not  at all | Several days | More than half the days | Nearly every day |
| --- | --- | --- | --- | --- |
| 1. Feeling nervous, anxious or on edge | 0 | 1 | 2 | 3 |
| 2. Not being able to stop or control worrying | 0 | 1 | 2 | 3 |
| 3. Worrying too much about different things | 0 | 1 | 2 | 3 |
| 4. Trouble relaxing | 0 | 1 | 2 | 3 |
| 5. Being so restless that it is hard to sit still | 0 | 1 | 2 | 3 |
| 6. Becoming easily annoyed or irritable | 0 | 1 | 2 | 3 |
| 7. Feeling afraid as if something awful   might happen | 0 | 1 | 2 | 3 |

**Column totals: ___ + ___ + ___ + ___**

***=* *Total Score _____***

**If you checked off any problems, how difficult have these problems made it for you to do your work, take care of things at home, or get along with other people?**

| **Not difficult  at all** | **Somewhat  difficult** | **Very difficult** | **Extremely difficult** |
| --- | --- | --- | --- |
|  |  |  |  |

GAD-7 total score for the seven items ranges from 0 to 21. This is calculated by assigning scores of 0, 1, 2, and 3, to the response categories of “not at all,” “several days,” “more than half the days,” and “nearly every day,” respectively.
