# Supplementary material for: Effectiveness of an unguided modular online intervention for highly anxious parents in preventing anxiety in their children: a parallel group randomised controlled trial
Source: Lancet Reg Health Eur. 2024 Sep 4;45:101038. doi: 10.1016/j.lanepe.2024.101038 (PMC11405817; doi:10.1016/j.lanepe.2024.101038)
Supplement: Supplementary Tables [file mmc3.docx]

**STATISTICAL ANALYSIS PLAN**

**Trial registration number**: <https://clinicaltrials.gov/ct2/show/NCT04755933>

**SAP version**: 2.0

**Protocol**: <https://www.researchprotocols.org/2022/11/e40707>

| **Persons contributing to the analysis plan** | |
| --- | --- |
| **Name** | Dr Chris Jones |
| **Position** | Senior Research Fellow in Medical Statistics  Brighton and Sussex Medical School |

| **Authorisation (v1.0)** | |
| --- | --- |
| **Name** | Prof Stephen Bremner |
| **Position** | Professor of Medical Statistics  Brighton and Sussex Medical School |
| **Signature** | 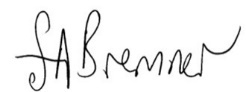 |
| **Date** | 26.5.23 |
|  |  |
| **Name** | Prof Sam Cartwright-Hatton |
| **Position** | Professor of Clinical Child Psychology  University of Sussex |
| **Signature** | 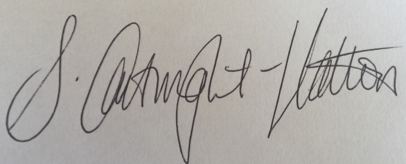 |
| **Date** | 26.5.23 |

| **Authorisation (v2.0)** | |
| --- | --- |
| **Name** | Prof Richard Emsley |
| **Position** | Professor of Medical Statistics and Trials Methodology.  Institute of Psychiatry, Psychology & Neuroscience |
| **Signature** | 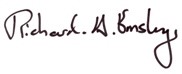 |
| **Date** | 7.7.23 |
|  |  |
| **Name** | Prof Stephen Bremner |
| **Position** | Professor of Medical Statistics  Brighton and Sussex Medical School |
| **Signature** |  |
| **Date** |  |
|  |  |
| **Name** | Prof Sam Cartwright-Hatton |
| **Position** | Professor of Clinical Child Psychology  University of Sussex |
| **Signature** |  |
| **Date** |  |

Contents

[1. SAP revision history 4](#_Toc139542913)

[2. Abbreviations 4](#_Toc139542914)

[3. Introduction 5](#_Toc139542915)

[3.1 Background and rationale 5](#_Toc139542916)

[3.2 Study objectives 5](#_Toc139542917)

[4. Study aims and objectives 5](#_Toc139542918)

[4.1 SAP versions 5](#_Toc139542919)

[4.2 Study design 5](#_Toc139542920)

[4.3 Randomisation 5](#_Toc139542921)

[4.4 Sample size 5](#_Toc139542922)

[4.5 Framework 6](#_Toc139542923)

[4.6 Statistical interim analyses and stopping guidance 6](#_Toc139542924)

[4.6.1 Interim analyses 6](#_Toc139542925)

[4.6.2 Early stopping guidelines 6](#_Toc139542926)

[4.7 Timing of final analysis 6](#_Toc139542927)

[4.8 Timing of outcome assessments 6](#_Toc139542928)

[5. Statistical principles 6](#_Toc139542929)

[5.1 Confidence intervals and p values 6](#_Toc139542930)

[5.2 Adherence and protocol deviations 6](#_Toc139542931)

[5.3 Analysis populations 7](#_Toc139542932)

[6. Study population 7](#_Toc139542933)

[6.1 Screening data 7](#_Toc139542934)

[6.2 Eligibility Criteria 7](#_Toc139542935)

[6.3 Recruitment 8](#_Toc139542936)

[6.4 Withdrawal/follow up 8](#_Toc139542937)

[6.5 Baseline participant characteristics 8](#_Toc139542938)

[7. Analysis 9](#_Toc139542939)

[7.1 Outcome definitions 9](#_Toc139542940)

[7.1.1 Parent, co-parent and other co-respondent outcomes 9](#_Toc139542941)

[7.1.2 Combining SCAS and SCAS-Pre scores 12](#_Toc139542942)

[7.2 Analysis methods 13](#_Toc139542943)

[7.2.1 Descriptive analysis 13](#_Toc139542944)

[7.2.2 Objective 1 13](#_Toc139542945)

[7.2.3 Moderators 14](#_Toc139542946)

[7.2.4 T3 data 15](#_Toc139542947)

[7.3 Objective 2 16](#_Toc139542948)

[7.3.1 Objective 2 aim 1 16](#_Toc139542949)

[7.3.2 Objective 2 aim 2 16](#_Toc139542950)

[7.3.3 Objective 2 aim 3 19](#_Toc139542951)

[7.4 Missing data 20](#_Toc139542952)

[7.4.1 Differential attrition sensitivity analysis 20](#_Toc139542953)

[7.4.2 MNAR sensitivity analysis 20](#_Toc139542954)

[7.5 Additional analyses 21](#_Toc139542955)

[7.6 Statistical software 21](#_Toc139542956)

[8. References 22](#_Toc139542957)

# SAP revision history

| **Version updated** | **Updated version number** | **Summary of changes** | **Author of changes** | **Date** |
| --- | --- | --- | --- | --- |
| Pre-1.0 | 1.0 | Authorisation of all SAP sections except those relating to objective 2 by study CI and senior statistician. | Chris Jones | 25/05/2023 |
| 1.0 | 2.0 | Authorisation of objective 2 section. | Chris Jones | 06/07/2023 |
|  |  |  |  |  |
|  |  |  |  |  |

# Abbreviations

| **Abbreviation** | **Meaning** |
| --- | --- |
| CACE | Complier Average Causal Effect |
| CPBQ | Challenging Parental Behaviour Questionnaire |
| EQ-5D-Y Proxy | EuroQol 5-dimension proxy measure for young persons |
| GAD7 | Generalised Anxiety Disorder assessment |
| MAR | Missing At Random |
| MNAR | Missing Not At Random |
| PWA | Parenting with Anxiety |
| SCAS | Spence Children’s Anxiety Scale |
| SCARED-A | Screen for Child Anxiety Related Emotional Disorders (Adult) |
| SEM | Structural Equation Modelling |
| SWAT | Study within a trial |
| SWEMWBS | Short Warwick and Edinburgh Mental Wellbeing Scale |

# Introduction

## Background and rationale

*See protocol paper*.

## Study objectives

*From protocol paper.*

# Study aims and objectives

This study aims to evaluate the effectiveness of a web-based intervention designed to prevent anxiety in the children of parents with anxiety and provide information for the optimization of this intervention. The study has 3 core objectives:

**Objective 1**: To investigate the effectiveness of a web-based, parent-focused intervention for the prevention of anxiety in children of parents with anxiety.

**Objective 2**: To determine which components of the intervention have the most or least impact on outcomes and test whether the effect of each component is moderated by participant characteristics (type or severity of parent or child anxiety symptoms, socioeconomic status, and child age).

**Objective 3**: To explore the impact of coparent anxiety and parenting behaviours on child outcomes—we hypothesize that child outcomes will be worse when the coparent is also anxious or engages in frequent anxiogenic parenting behaviours.

## SAP versions

This SAP will cover the analyses relating to the comparison arms and intervention (objectives 1 and 2). A separate SAP and analysis will be developed for objective 3. Version 1.0 of this SAP contains all analyses except for those for objective 2. Draft analyses for objective 2 are included and will be confirmed in version 2.0 of the SAP.

## Study design

A non-blinded randomised controlled trial (intervention vs control).

## Randomisation

1:1 permuted block randomisation to intervention or control with random block sizes. Participants’ randomisations were assigned at sign up (prior to consent) but participants were only considered part of the study after they had consented and been informed of their randomisation after completion of baseline measures. This reduced the balancing effect of the blocking but would not be expected to have any meaningful effect on balance between the arms.

For the intervention arm only, participants were further randomised to one of eight groups (1:1:1:1:1:1:1:1) each excluding one of the intervention modules. The order of the seven retained modules was also random.

## Sample size

*From protocol paper.*

The sample size was calculated to provide adequate power for our first objective (to detect a difference between trial arms in child anxiety). Based on existing research carried out by the
trial team, a small effect size is anticipated (Cohen d=0.2) [17]. With 90% power for 5% significance, this requires 526 participants in each of 2 arms of the trial. Allowing 40% attrition, which is likely to be substantial in a web-based study, we need to randomize 877 participants to each arm, or 1754 in
total.

## Framework

Superiority.

## Statistical interim analyses and stopping guidance

### Interim analyses

No interim analyses were planned or performed.

### Early stopping guidelines

n/a

## Timing of final analysis

Final analysis will be conducted after completion of second follow up (m9-25 depending on sign up date). Analysis is expected to begin in May 2023.

## Timing of outcome assessments

Outcomes were collected at baseline, m6 and m9-m25. Completion of outcomes relative to each time point will be visualised graphically.

# Statistical principles

## Confidence intervals and p values

95% confidence intervals will be reported. p-values will be interpreted in terms of strength of evidence against the null hypothesis of no difference between the arms.

## Adherence and protocol deviations

Adherence to intervention will be quantified per module as percentage of participants assigned to that module completing at least 90%. The percentage of participants completing at least 90% of 3 or more modules will also be summarised.

## Analysis populations

Analyses will be performed following intention to treat principles. The study data consists of two populations: parents and co-respondents. The analyses for objectives 1 and 2 apply to the parent population and the analysis of objective 3 applies to parents with a corresponding co-respondent. Co-respondents can be co-parents or others. Where relevant, co-respondents are referred to as “all co-respondents”, “co-parents only”, or “other co-respondents”. Parents, co-parents and other co-respondents complete different sets of outcomes (with some overlap).

# Study population

## Screening data

Numbers of signed up, eligible, consented and participants informed of their randomisation after completing baseline measures will be reported.

## Eligibility Criteria

*From protocol paper.*

The eligibility criteria are designed to resemble those that would be employed in any eventual rollout of a web-based intervention. As such, they are minimal. To be eligible, a participant must be a parent (any gender; adoptive, biological, step, foster, or grandparent) residing in the United Kingdom, aged over 16 years and with a child aged 2 to 11 years (inclusive). The participant must have at least 50 days’ contact with the index child per year and confirm that they see enough of the child to report on the child’s current anxiety level. The participant must self-report subjectively substantial levels of current or lifetime anxiety (it is not necessary to have a diagnosis) and be able to commit to completion of measures at (up to) 3 time points, even if allocated to the control arm.

Participants will not be excluded on the basis of current or previous psychiatric treatment (parent or child) or on any psychological, neuropsychological, or physical condition.

## Recruitment

Participant progress through the study will be summarised using a CONSORT flow diagram (Schulz *et al.*, 2010)

## Withdrawal/follow up

Numbers withdrawing and reasons for withdrawal will be summarised by arm.

## Baseline participant characteristics

For parents, the flowing baseline characteristics will be summarised:

- Birth gender (birthgender_demog, Male/Female/Prefer not to say)
- Age (user_age, continuous)
- Ethnicity (ethnicity_demog, multiple categories)
- Financial status (financialstatus_demog, Comfortable/Managing/Struggling)
- Previous treatment for anxiety within last 12 months (ptpanxietytx_t1_demog, No/Yes)
- Education (education_demog, multiple categories)
- Number of children (nchildren, integer)
- Lives with other parent of index child (pstatus_pexp, No/Yes)

For index children, the flowing baseline characteristics will be summarised:

- Gender (indexchildgender_ch)
- Age (age_ch)
- Ethnicity (indexchildethnic_ch, multiple categories)
- Developmental disability (childdevdis_ch, No/Yes)
- Previous treatment for anxiety within last 12 months (ChildAnxietyTx_T1Q88, No/Yes)

For co-respondents, the following characteristics will be summarised:

- Birth gender (birthgender_demog, Male/Female/Prefer not to say)
- Age (age, continuous)
- Ethnicity (ethnicitycp_cop and ethnicitycr_cor, multiple categories)
- Financial status (financialstatuscp_cop and financialstatuscr_cor, Comfortable/Managing/Struggling)
- Education (educationcp_cop and educationcr_cor, multiple categories)
- Relationship to parent (friend/grandparent/other/parent/relation)

# Analysis

## Outcome definitions

### Parent, co-parent and other co-respondent outcomes

**Table 1:** Outcomes

| **Outcome** | **Subscales** | **Scoring** | **Missing data** |
| --- | --- | --- | --- |
| **SCAS**  0=Never  1=Sometimes  2=Often  3=Always  38 questions  Completed by parents, co-parents, and other co-respondents.  Quantifies child anxiety (ages 6 to 18).  Higher scores indicate greater child anxiety. | Panic and agoraphobia | Sum items Q12 Q19 Q25 Q27 Q28 Q30 Q32 Q33 Q34 | Missing values will be prorated if ≥80% of the subscale is complete. Mean of available data within subscales will be calculated and rounded to the nearest integer. |
|  | Separation anxiety | Sum items Q5 Q8 Q11 Q14 Q15 Q38 |  |
|  | Physical injury fears | Sum items Q2 Q16 Q21 Q23 Q29 |  |
|  | Social phobia | Sum items Q6 Q7 Q9 Q10 Q26 Q31 |  |
|  | Obsessive compulsive | Sum Q13 Q17 Q24 Q35 Q36 Q37 |  |
|  | Generalised anxiety | Sum items Q1 Q3 Q4 Q18 Q20 Q22 |  |
|  | Overall score | Sum of all subscales (max score 114) | Score will only be calculated if all subscale scores are available. |
| **SCAS-P**  0=Not true at all  1=Seldom true  2=Sometimes true  3=Quite often true  4=Very often true  28 questions  Completed by parents, co-parents, and other co-respondents.  Quantifies child anxiety (ages 3 to 6).  Higher scores indicate greater child anxiety. | Separation anxiety | Sum items Q6 Q12 Q16 Q22 Q25 | Missing values will be prorated if ≥80% of the subscale is complete. Mean of available data within subscales will be calculated and rounded to the nearest integer. |
|  | Physical injury fears | Sum items Q7 Q10 Q13 Q17 Q20 Q24 Q26 |  |
|  | Social anxiety | Sum items Q2 Q5 Q11 Q15 Q19 Q23 |  |
|  | Obsessive compulsive disorder | Sum Q3 Q9 Q18 Q21 Q27 |  |
|  | Generalised anxiety | Sum items Q1 Q4 Q8 Q14 Q28 |  |
|  | Overall score | Sum of all subscales (max score 112) | Score will only be calculated if all subscale scores are available. |
| **Standardised SCAS**  (Parents, co-parents and other co-respondents)  Higher scores indicate greater child anxiety. | Overall | SCAS and SCAS-P overall scores will be standardised (per time point) to allow combination for modelling (see below) | N/A. |
| **PSC-17**  0=Never  1=Sometimes  2=Often  17 questions (+1 additional No/Yes question which is not included in scoring)  Completed by parents.  Quantifies child anxiety.  Higher scores indicate greater child anxiety. | Internalising | Sum items 1 to 5 | Items that are left blank are scored 0. If four or more items are left blank, overall score will not be calculated.  Subscale scores will be calculated (with missing values treated as 0) if ≥80% complete. |
|  | Attention | Sum items 6 to 10 |  |
|  | Externalising | Sum items 11 to 17 |  |
|  | Overall score | Sum items 1 to 17 |  |
| **EQ-5D-Y Proxy**  No problems/pain/worry  Some problems/ pain/worry  A lot of problems/pain/worry  5 questions + Visual Analogue Scale  Completed by parents.  Quantifies child Quality of Life.  Higher scores indicate lower child QoL, except on the VAS, where higher scores indicate greater child QoL. |  | Individual questions and VAS will be summarised separately. There is no UK value set available, so index score will not be calculated. | N/A (complete data reported for individual items). |
| **CPBQ**  1=not applicable  2=Somewhat applicable  3=Sometimes applicable and sometimes not applicable  4=Usually applicable 5=Completely applicable  104 questions  Completed by parents and co-parents.  Quantifies child behaviours.  Higher scores indicate greater parent behaviour.  Note: Question numbers for subscales reflect database numbering. | Challenging behaviour | Sum items Q1 Q37 Q69 Q91 Q7 Q43R Q73 Q94 Q13 Q49 Q77 Q97 Q19 Q54 Q81 Q100 Q104 Q25 Q59 Q85 Q102 Q31 Q64 Q88 Q103 | Missing values will be prorated if ≥80% of the subscale is complete. Mean of available data within subscales will be calculated and rounded to the nearest integer. |
|  | Overinvolvement | Sum items Q2 Q26 Q50 Q70 Q14 Q38 Q60 Q78 Q8 Q44 Q74 Q89 Q20 Q55 Q82 Q92 Q98 Q32R Q65R Q86R Q95R |  |
|  | Warmth | Sum items Q3 Q27 Q51 Q71 Q9 Q33 Q56 Q75 Q15 Q39 Q61 Q79R Q21 Q45 Q66 Q83 |  |
|  | Negativity | Sum items Q4 Q16 Q28 Q40 Q10 Q22 Q34 Q46R |  |
|  | Negative discipline | Sum items Q5 Q41 Q72 Q93 Q52 Q17 Q80 Q99 Q29 Q62 Q87 Q101R Q11 Q35 Q57 Q90 Q76 Q23 Q47 Q67 Q84 Q96R |  |
|  | Positive discipline | Sum items Q6 Q24 Q42 Q58 Q12 Q30 Q48 Q63 Q18 Q36 Q53 Q68 |  |
| **SCARED-A**  0=Almost never  1=Sometimes  2=Often  71 questions  Completed by parents and co-parents.  Quantifies parent anxiety.  Higher scores indicate greater parent anxiety. | Panic disorder | Sum items Q1 Q9 Q14 Q18 Q28 Q34 Q37 Q43 Q47 Q51 Q54 Q59 Q64 | Missing values will be prorated if ≥80% of the subscale is complete. Mean of available data within subscales will be calculated and rounded to the nearest integer. |
|  | Generalised anxiety disorder | Sum items Q8 Q11 Q21 Q41 Q44 Q52 Q58 Q60 Q62 |  |
|  | Social phobia | Sum items Q4 Q15 Q24 Q33 Q39 Q50 Q56 Q63 Q71 |  |
|  | Separation anxiety disorder | Sum items Q3 Q7 Q13 Q17 Q19 Q30 Q31 Q38 Q48 Q53 Q55 Q61 |  |
|  | Obsessive-compulsive disorder | Sum items Q6 Q10 Q12 Q25 Q27 Q32 Q42 Q57 Q66 |  |
|  | Post traumatic stress disorder | Sum items Q26 Q46 Q49 Q68 |  |
|  | Phobias | Sum of Q22 Q40 Q69 Q5 Q16 Q20 Q35 Q36 Q45 Q70 Q2 Q23 Q29 Q65 Q67 |  |
|  | Overall score | Sum of all subscales (max score 142) | Score will only be calculated if all subscale scores are available. |
| **SWEMWBS**  1=None of the time  2=Rarely  3=Some of the time  4=Often  5=All of the time  7 questions  Completed by parents.  Quantifies parent mental wellbeing.  Higher scores indicate greater parent mental wellbeing. | Overall score | Sum all 7 items (max score 35), convert to metric score using  <https://warwick.ac.uk/fac/sci/med/research/platform/wemwbs/using/howto/swemwbs_raw_score_to_metric_score_conversion_table.pdf> | Missing values will be prorated if ≥80% of the subscale is complete. Mean of available data will be calculated and rounded to the nearest integer. |

| **GAD7**  0=Not at all sure  1=Several days  2=Over half the days  3=Nearly every day  7 questions  Completed by other co-respondents.  Quantifies co-respondent anxiety.  Higher scores indicate greater co-respondent anxiety. | Overall score | Sum all 7 items (max score 21) | Missing values will be prorated if ≥80% of the subscale is complete. Mean of available data will be calculated and rounded to the nearest integer. |
| --- | --- | --- | --- |

All outcomes are collected at baseline, T2 and T3.

### Combining SCAS and SCAS-Pre scores

The SCAS and SCAS-Pre outcomes have different numbers of questions, response scales and overall scores. To allow them to be analysed together, standardised z-scores will be calculated for SCAS and SCAS-P overall scores. z-scores will be calculated separately for SCAS and SCAS-Pre as z=(x_it_-m_t_)/sd_t_ at each time point, where x_it_ is the value of SCAS/SCAS-Pre for the *i*th individual at time point *t* and m_t_ and sd_t_ are respectively the mean and SD of SCAS/SCAS-Pre at time point *t*.

## Analysis methods

### Descriptive analysis

Descriptive statistics will be reported by randomization arm for participant demographics and all parent outcomes/subscales listed in Table 1 at baseline, using appropriate summary statistics (counts and percentages for binary and categorical variables and means and SDs or medians with lower and upper quartiles for continuous variables).

Number of adverse events will be presented as the number of events and number of individuals with events by randomisation arm and in accordance with the treatment received. We will also report the distribution of the change in parent anxiety (T2-T1) and number of parents in each arm whose anxiety worsens (based on SCARED-A overall score).

The distress, effects and evaluation questions (regarding participation in the study) collected at T2 will be summarised by intervention arm.

### Objective 1

Multiple linear regression models will be used to assess the effect of the intervention on the following child outcomes at T2:

- Standardised SCAS/SCAS-Pre overall score (primary outcome)
- PSC Internalising subscale score
- PSC Attention subscale score
- PSC Externalising subscale score

And on parent outcomes:

- SCARED-A overall score

These models will include a fixed effect for intervention arm. The following covariates considered to be prognostic of outcome will be included as fixed effects in the models:

- Outcome at baseline
- Child anxiety at baseline (SCAS, redundant in SCAS model)
- Parent anxiety (SCARED-A, redundant in SCARED-A model)
- Parent gender (birthgender, Male/Female/Prefer not to say or missing)
- Child Birth gender (indexchildgender, Male/Female/Prefer not to say or missing)
- Child Age (child_age, continuous)

Treatment effects (between-group differences) will be reported as adjusted mean difference with 95% confidence intervals. Cohen’s D effect sizes at 6 months will be calculated as adjusted mean difference of outcome divided by sample standard deviation of the outcome at baseline.

### Moderators

Potential moderators will be assessed by repeating the SCAS model including intervention arm by moderator interactions as fixed effects (in separate models). This analysis will not be performed if <5% of participants are represented in one category (for binary variables). Categories will be merged if there is <5% representation in any of the categories. The moderators to be assessed are:

- Severity of child anxiety at baseline (SCAS)
- Panic and agoraphobia anxiety on SCAS at baseline (defined below)
- Separation anxiety on SCAS/SCAS-P at baseline (defined below)
- Social phobia on SCAS/SCAS-P at baseline (defined below)
- Obsessive compulsive on SCAS/SCAS-P at baseline (defined below)
- Generalised anxiety on SCAS/SCAS-P at baseline (defined below)
- Severity of parent anxiety at baseline (SCARED-A, defined below)
- Panic disorder anxiety on SCARED-A at baseline (defined below)
- Generalised anxiety disorder anxiety on SCARED-A at baseline (defined below)
- Social phobia anxiety on SCARED-A at baseline (defined below)
- Separation anxiety disorder anxiety on SCARED-A at baseline (defined below)
- Obsessive-compulsive disorder anxiety on SCARED-A at baseline (defined below)
- Post traumatic stress disorder anxiety on SCARED-A at baseline (defined below)
- Phobias anxiety on SCARED-A at baseline (defined below)
- Change in parent anxiety between T1 and T2 (Overall SCARED-A)
- Socioeconomic status (FinancialStatus variable (comfortable/managing/struggling))
- Parent gender (birthgender)
- Child gender (indexchildgender)
- Child age (child_age, continuous)
- Child developmental disabilities (childdevdis, No/Yes)

**SCAS subscale anxiety definitions**

Definitions of anxiety based on each of the SCAS/SCAS-P subscales (except Physical injury fears) will be based on population norms: <https://www.scaswebsite.com/portfolio/scas-parent-norms/> and <https://www.scaswebsite.com/portfolio/scas-preschool-norms_and_interpretation/>. Scores 1.65 (representing the top 10% of expected scores) standard deviations above the mean will be considered positive for each subscale.

**SCARED-A anxiety definitions**

Definitions of anxiety based on each of the SCARED-A subscales will be based on population norms: <https://www.researchgate.net/profile/Fja-bonny-Van-Steensel/publication/263057747_An_adult_version_of_the_Screen_for_Child_Anxiety_Related_Emotional_Disorders_SCARED-A/links/0c960539ad128cec17000000/An-adult-version-of-the-Screen-for-Child-Anxiety-Related-Emotional-Disorders-SCARED-A.pdf> (Table 5, non-clinical population). Scores 1.65 (representing the top 10% of expected scores) standard deviations above the mean will be considered positive for each subscale.

Overall anxiety on SCARED-A will be defined as a score of ≥20 for males and ≥30 for females.

### T3 data

The outcome models for Objective 1 will be extended to include T3 data by including a fixed effect for time, and a random effect for participant.

## Objective 2

Objective 2 will include three analyses with the following aims:

1. To assess the effect of intervention with/without each module A-H on child outcomes.

2. To assess the effect of intervention with/without each module A-H on mediator outcomes (parent behaviour outcomes made up of questions relating to the respective module).

3. To perform mediation analyses to assess the effect of the intervention on child outcomes directly and through each mediator outcome.

These analyses will be conducted for child primary outcomes (Standardised SCAS score and PSC-17 Internalising, Attention, and Externalising subscale scores) for which an effect has been observed in the primary analyses for objective 1.

### Objective 2 aim 1

For modules A-H, a multiple linear regression model will be fitted for each child outcome at T2 in the same way as for the primary outcomes, except with Intervention (Intervention/Control) replaced with Intervention split into three groups: Intervention without the module, Intervention with the module, and Control.

The effect estimates, effect sizes, 95% CIs and p-values will be reported for:

- Intervention with the module vs Control
- Intervention without the module vs Control
- Intervention with the module vs Intervention without the module

### Objective 2 aim 2

For modules A-H, a multiple linear regression model will be fitted for the relevant mediator outcome at T2 in the same way as for the primary outcomes, except with Intervention (Intervention/Control) replaced with Intervention split into three groups: Intervention without the module, Intervention with the module, and control.

The effect estimates, effect sizes, 95% CIs and p-values will be reported for:

- Intervention with the module vs Control
- Intervention without the module vs Control
- Intervention with the module vs Intervention without the module

For the core module, a multiple linear regression model will be fitted for the core mediator outcome as above, but with intervention grouped as Intervention/Control, as all intervention participants complete this module.

Table 2 shows which questions make up the mediator outcomes and to which module mediator each relates. Mediator outcomes will be scored as the mean of the responses to the constituent questions as long as at least 80% of the questions are complete. Completeness will be calculated after prorating of missing values for the CPBQ questions as described in Table 1.

All questions, except those for module E, are on the scale 1-5. Questions for module E, on the scale 1-4 will be rescaled to match (1=1, 2=2.33, 3=3.67, 4=5).

**Table 2:** Modules and their respective mediators.

| **Module** | **Mediator** | **Questions** |
| --- | --- | --- |
| Core | Mediator Core | mediator_core_1 (1-5)  mediator_core_2 (1-5)  mediator_core_3 (1-5)  mediator_core_4 (1-5)  mediator_core_5 (1-5)  mediator_core_6 (1-5)  mediator_core_7 (1-5)  mediator_core_8 (1-5)  mediator_core_9 (1-5) |
| Topic A  Avoidance  3 questions | Mediator A | CPBQ Q13 (1-5)  mediator_a_1 (1-5)  mediator_a_2 (1-5) |
| Topic B  Play  9 questions | Mediator B | CPBQ Q3 (1-5)  CPBQ Q51 (1-5)  CPBQ Q7 (1-5)  CPBQ Q43 (1-5, reverse scored)  CPBQ Q73 (1-5)  CPBQ Q94 (1-5)  CPBQ Q77 (1-5)  mediator_b_1 (1-5)  mediator_b_2 (1-5) |
| Topic C  Emotion coaching  3 questions | Mediator C | mediator_c_1 (1-5)  mediator_c_2 (1-5)  mediator_c_3 (1-5) |
| Topic D  Positive behaviour management  8 questions | Mediator D | CPBQ Q30 (1-5)  CPBQ Q63 (1-5)  CPBQ Q12 (1-5)  CPBQ Q48 (1-5)  mediator_d_1 (1-5)  mediator_d_2 (1-5)  mediator_d_3 (1-5)  mediator_d_4 (1-5) |
| Topic E  Basic needs/hacks  4 questions | Mediator E | mediator_e_1 (1-4)  mediator_e_2 (1-4)  mediator_e_3 (1-4)  mediator_e_4 (1-4) |

**Table 2** cont.

| Topic F  Hotspots and overprotection  8 questions | Mediator F | mediator_f_1 (1-5)  CPBQ Q2 (1-5)  CPBQ Q26 (1-5)  CPBQ Q50 (1-5)  CPBQ Q70 (1-5)  mediator_f_2 (1-5)  mediator_f_3 (1-5)  mediator_f_4 (1-5) |
| --- | --- | --- |
| Topic G  Modelling and compensation  4 questions | Mediator G | CPBQ Q103 (1-5)  mediator_g_1 (1-5)  mediator_g_2 (1-5)  mediator_g_3 (1-5) |
| Topic H  Limit setting and consequences  16 questions | Mediator H | CPBQ Q11 (1-5)  CPBQ Q35 (1-5)  CPBQ Q57 (1-5)  CPBQ Q90 (1-5)  CPBQ Q76 (1-5)  CPBQ Q23 (1-5)  CPBQ Q47 (1-5)  CPBQ Q84 (1-5)  CPBQ Q96 (1-5)  CPBQ Q36 (1-5)  CPBQ Q53 (1-5)  CPBQ Q68 (1-5)  CPBQ Q5 (1-5)  CPBQ Q41 (1-5)  CPBQ Q93 (1-5)  CPBQ Q99 (1-5) |

### Objective 2 aim 3

For each module/mediator, we will perform a mediation analysis by Structural Equation Modelling, with the child outcome at T2 as the dependent variable, intervention as a predictor, and the measure of parenting behaviour congruent with that module as a mediator. Each model will include all participants and intervention will be grouped as Intervention with module, Intervention without module, and Control (Figure 1).

Direct, indirect and total effects on outcome at T2 for each intervention group will be reported with bootstrapped 95% CIs.

**Figure 1:** SEM mediation analysis for each module.


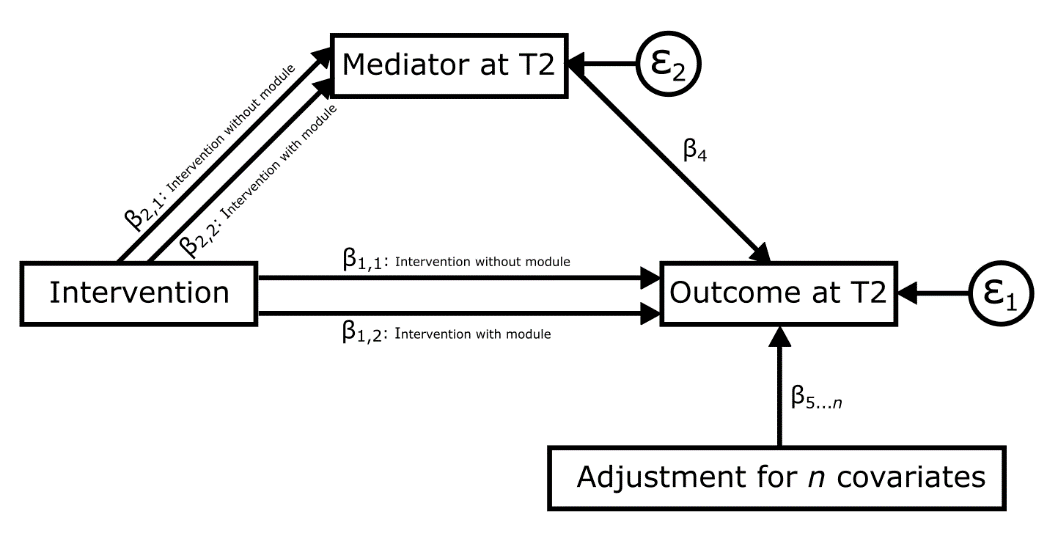


## Missing data

It is known that that there is differential attrition between arms of the PWA study and the primary analyses will be performed for complete cases, under the assumption that data were missing at random (MAR). It is possible that complete case analysis with differential attrition under the MAR assumption can lead to biased effect estimates (Bell 2013). Potentially more significantly, if the MAR assumption is not valid and data are missing not at random (MNAR), e.g. if participants with low outcome score are more likely to be missing, then the estimates will be biased by an unknown amount. It is not possible to statistically determine if data are MNAR, but the robustness of the analyses performed to deviations from the MAR assumption can be examined by varying assumptions about the missing values.

The following sensitivity analyses will be conducted to assess the effect of differential attrition under the MAR assumption and in combination with violation of the MAR assumption.

### Differential attrition sensitivity analysis

The primary outcome models will be refitted using full information maximum likelihood (using SEM), where observations with missing data are not dropped from the analysis and all available data is used in the model. Estimates of intervention effects with their 95% CIs will be reported for comparison to the primary analysis results to assess the extent of any bias due to differential attrition between arms.

### MNAR sensitivity analysis

The Stata module RCTMISS will be used to assess the effect on the primary analysis results under different assumptions about the missing data. Departures from MAR will be expressed as a pre-defined value of sensitivity parameter delta, which represents the average difference between observed and unobserved values of the outcome. Delta will be applied to replace missing data in both arms, and in one arm at a time. The intervention effect estimates and their 95% CIs under different values of delta will be reported and presented visually.

The following values of delta will be used for each primary outcome:

- Standardised SCAS score: -2 to +2 (standard deviations) in increments of 0.25 (roughly equal to 4 points on the original scales).
- PSC Internalising subscale score (range 0 to 10): -3 to +3 in increments of 0.5.
- PSC Attention subscale score (range 0 to 10): -3 to +3 in increments of 0.5.
- PSC Externalising subscale score (range 0 to 14): -4 to +4 in increments of 0.5.
- SCARED-A overall score (range 0 to 142): -60 to +60 in increments of 10.

Note: delta values listed above assume outcome averages are mid-range and may need to be adjusted if this is not the case.

## Additional analyses

Complier average causal effect (CACE) analyses will be conducted to assess the effect of the intervention in participants in the intervention arm that complied with the intervention and those in the control arm who would have complied with the intervention if they had had the opportunity to do so.

Three CACE analyses will be conducted with compliance defined as:

- Participants who have accessed more than 2 modules (>0% completion of at least 3 modules.
- Participants who have completed more than 2 modules (>2 modules ≥90% complete)
- Participants who have completed all modules (8 modules ≥90% complete).

The CACE analyses will be conducted using Structural Equation Modelling.

## Statistical software

Stata version 17.0 or later will be used for analysis.

# References

Bell ML, Kenward MG, Fairclough DL, Horton NJ. (2013) Differential dropout and bias in randomised controlled trials: when it matters and when it may not. BMJ 346:e8668

Schulz KF, Altman DG, Moher D, for the CONSORT Group. (2010) CONSORT 2010 Statement: updated guidelines for reporting parallel group randomised trials. Trials 11:32.
